# A fibril-selective single-domain antibody targets pathogenic amyloid-β assemblies and protects human neurons

**DOI:** 10.1016/j.isci.2026.117946

**Published:** 2026-10-21

**Authors:** Matt A. Greenlee, Soukaina Amniouel, Isabel Rivera, Jessica Suh, Natalia J. Martinez, Anton Simeonov, Elizabeth Head, Robert Tycko, Wei Zheng, Bryan D. Fleming, Qi Zhang

**Affiliations:** 1 Early Translation Branch, National Center for Advancing Translational Sciences, National Institutes of Health, Rockville, MD 20850, USA; 2 Therapeutic Development Branch, National Center for Advancing Translational Sciences, National Institutes of Health, Rockville, MD 20850, USA; 3 Department of Pathology and Laboratory Medicine, University of California, Irvine, Irvine, CA 92697, USA; 4 Laboratory of Chemical Physics, National Institute of Diabetes, Digestive, and Kidney Diseases, National Institutes of Health, Bethesda, MD 20892, USA

**Keywords:** Alzheimer disease, single-domain antibody, amyloid-β fibrils, human brain organoids, resource tool

## Abstract

Alzheimer disease is characterized by extracellular amyloid-β deposition that contributes to neuronal dysfunction and degeneration. Although anti-amyloid-β antibodies reduce plaque burden, their use requires monitoring for amyloid-related imaging abnormalities. Here, phage display against immobilized Aβ40 fibrils representing a patient-relevant polymorph identified J8, a fibril-selective, Fc-free single-domain antibody. J8 bound fibrils with high apparent affinity, showed no detectable binding to monomeric Aβ40, decorated fibril surfaces, and recognized plaque-associated amyloid in Alzheimer disease brain tissue. During concurrent fibril exposure, J8 reduced apoptotic signaling in human induced pluripotent stem cell (iPSC)-derived cortical neurons and forebrain organoids. Single-nucleus profiling of pooled organoids identified fibril-associated stress programs and opposite-direction expression patterns in the FibJ8 condition; fibril-associated changes showed the strongest directional concordance with advanced disease pathology. These findings identify J8 as a fibril-selective candidate and support organoid-based evaluation of anti-amyloid reagents as a fit-for-purpose preclinical tool.

## Highlights

• J8 is a fibril-selective, Fc-free single-domain antibody sparing monomeric amyloid-β
• J8 decorates fibril surfaces and binds plaque-like deposits in human AD brain tissue
• J8 lowers fibril-induced apoptotic signaling in human neurons and organoids
• snRNA-seq shows J8 blunts fibril stress programs concordant with human AD

## Graphical abstract

**Figure:**
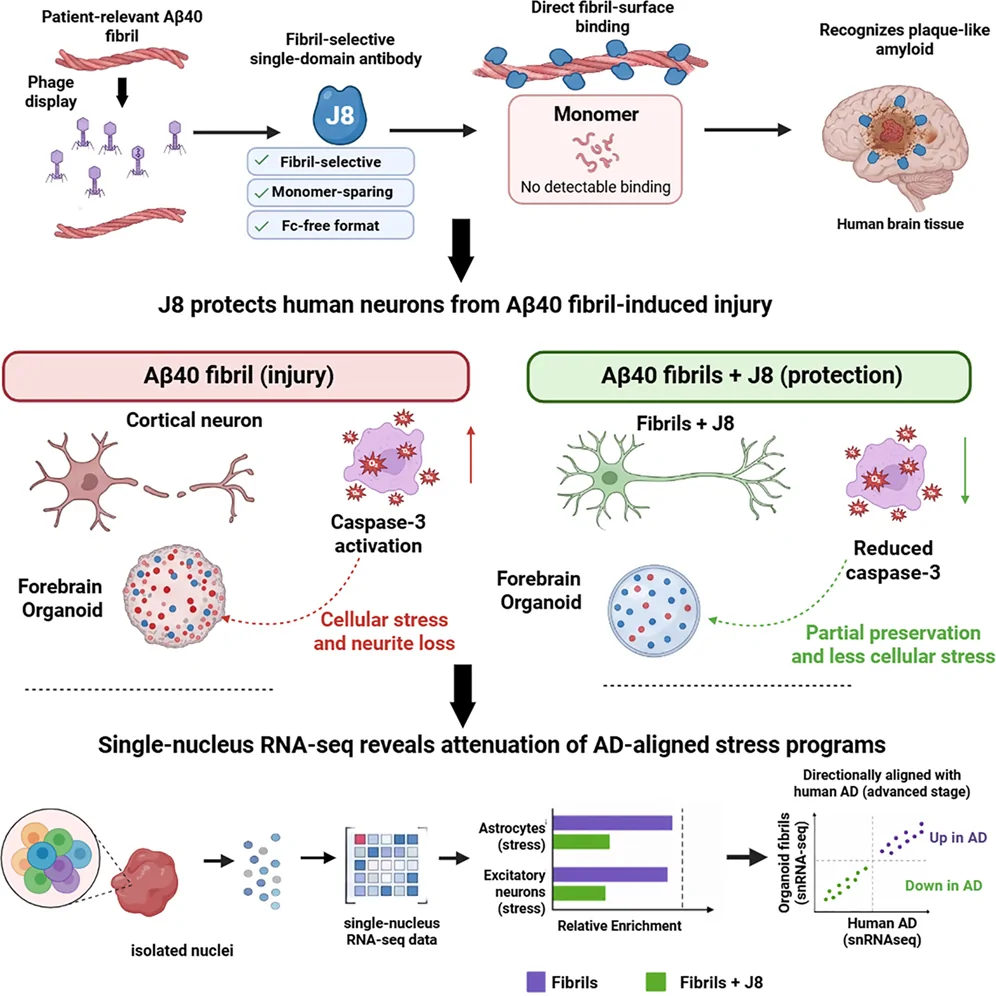

## Teaser

Neuroscience; Cell biology

## Introduction

Alzheimer disease (AD) is a progressive neurodegenerative disorder hallmarked pathologically by extracellular amyloid-β (Aβ) deposition, intracellular Tau pathology, and chronic neuroinflammation.^1^ Therapeutic strategies that target Aβ deposits have begun to demonstrate disease-modifying activity in early AD patients; monoclonal antibodies including aducanumab, lecanemab, and donanemab reduce amyloid plaques and confer modest clinical benefit in selected patient populations.^2^^,^^3^ Despite this progress, current immunoglobulin G (IgG)-based anti-amyloid therapies have a narrow therapeutic window and require intensive monitoring, largely due to amyloid-related imaging abnormalities (ARIAs) and other dose-limiting toxicities.^4^

ARIA includes vasogenic edema and sulcal effusions (ARIA-E), as well as hemosiderin-associated changes such as cerebral microhemorrhages and superficial siderosis (ARIA-H).^5^^,^^6^ Clinical experience indicates that ARIA risk is increased at higher therapeutic doses and in ApoE ε4 carriers, motivating stepwise dose escalation, MRI surveillance, and treatment interruptions in many clinical protocols.^7^^,^^8^^,^^9^^,^^10^^,^^11^ These constraints can limit treatment access and may restrict the dose levels or duration of therapy required to achieve sustained engagement of brain Aβ fibrils.

Aβ exists in multiple assembly states, including monomers, soluble oligomers, protofibrils, and mature fibrils, which differ in structure, stability, and biological activity.^12^^,^^13^ Aβ42 has been strongly implicated in AD because of its high aggregation propensity and the neurotoxicity attributed to soluble Aβ42 oligomeric and protofibrillar assemblies. Nevertheless, Aβ fibrils exhibit molecular-level polymorphism, and the disease relevance of an Aβ assembly may depend not only on peptide length but also on the conformation adopted in brain tissue. In a solid-state nuclear magnetic resonance study of Aβ40 and Aβ42 fibrils propagated by seeded growth from 37 cortical tissue samples obtained from 18 individuals with typical AD, posterior cortical atrophy AD, or rapidly progressive AD, Qiang et al. identified a single predominant Aβ40 fibril structural signature in most typical AD and posterior cortical atrophy samples. In contrast, rapidly progressive AD-derived Aβ40 fibrils contained a greater contribution from additional structures, and brain-seeded Aβ42 fibrils were structurally heterogeneous across the clinical groups.^14^ Ghosh et al. subsequently determined a 2.8 Å cryo-EM structure of this prevalent brain-seeded Aβ40 fibril polymorph. The structure displayed an unusual four-layered cross-β architecture with 2-fold screw symmetry, fully extended Aβ40 molecules in the two central layers, and density consistent with β-hairpin conformations in the outer layers.^15^ This organization is qualitatively distinct from the paired, S-shaped protofilament folds subsequently reported for Aβ42 filaments extracted directly from human brain tissue.^16^

Fibrillar Aβ constitutes the core of amyloid plaques and provides a persistent scaffold that can drive local cellular stress responses and seed further aggregation.^17^^,^^18^ In contrast, currently approved or late-stage anti-Aβ antibodies such as lecanemab and donanemab recognize broader aggregated Aβ species, including soluble protofibrils or plaque-associated epitopes, rather than selectively targeting fibrillar assemblies.^1^^,^^19^ While these broader binding profiles may facilitate plaque clearance, they may also engage heterogeneous Aβ species and associated immune responses. These considerations raise the possibility that more selective targeting of fibrillar Aβ could enable focused engagement of pathological deposits while minimizing off-target interactions with non-fibrillar Aβ species.

A substantial component of ARIA risk is thought to arise from the binding profiles and effector functions of full-length IgG antibodies.^20^^,^^21^ Antibodies that bind aggregated Aβ can engage Fc-dependent pathways, including complement activation and Fcγ receptor signaling on myeloid cells, which can amplify local inflammation around amyloid deposits in vessel walls and brain parenchyma. This Fc-driven inflammatory response is a plausible contributor to ARIA in the setting of cerebral amyloid angiopathy and anti-amyloid immunotherapy.^10^^,^^22^^,^^23^^,^^24^ Strategies that preserve pathological Aβ engagement while minimizing Fc-driven immune activation could therefore improve the therapeutic index of anti-amyloid immunotherapy.

Single-domain antibodies offer a rational format to address these limitations at the design level. At ∼15 kDa, they are much smaller than full-length IgG molecules and can diffuse more readily within brain parenchyma.^25^ Single-domain antibodies lack an Fc region and therefore do not directly engage complement or Fcγ receptors through an antibody Fc domain. However, the absence of an Fc region may also reduce microglial engagement and Fc-dependent clearance of antibody-bound amyloid. These molecules may also be selected for conformational epitopes, enabling preferential binding to fibrillar Aβ assemblies while sparing monomeric Aβ. Although Fc elimination alone does not guarantee ARIA avoidance, this format enables mechanistically informed lead selection and optimization before *in vivo* safety evaluation. In addition, single-domain antibodies provide engineering flexibility that is not readily achievable with conventional monoclonal antibodies. These molecules can be optimized for controlled brain delivery through receptor-mediated transcytosis or intranasal administration, allowing lower systemic exposure while maintaining therapeutic brain concentrations.^26^^,^^27^ Single-domain antibodies can also serve as targeting modules for conjugated payloads, enabling localized delivery of therapeutic agents to amyloid-rich regions without widespread immune activation.^28^ Together, these properties support investigation of single-domain antibodies as an alternative anti-amyloid format, while their effects on amyloid clearance, efficacy, and safety remain to be established.

Currently, there is growing interest in human-relevant new approach methodologies (NAMs) to complement conventional preclinical models in drug development. Recent Food and Drug Administration (FDA) draft guidance emphasizes that fit-for-purpose NAMs should be defined by a clear context of use, supported by human biological relevance, and technically characterized for robustness and reproducibility. In this setting, human forebrain organoids may help fill an important preclinical data gap by providing multicellular, human-relevant readouts of Aβ fibril-associated neural injury and treatment response. Using patient-relevant Aβ fibrils, postmortem AD brain tissue, human induced pluripotent stem cell (iPSC)-derived cortical neurons, and forebrain organoids with single-nucleus transcriptomic readouts, we evaluated whether a fibril-selective, Fc-free nanobody could achieve pathological target engagement and attenuate fibril-associated cellular injury in human-relevant systems.

Here, we report development of a single-domain antibody, J8, identified by phage display against immobilized Aβ40 fibrils that resemble a prevalent patient-derived fibril polymorph. We show that J8 binds to Aβ40 fibrils with high apparent affinity and exhibits no detectable binding to monomeric Aβ40. Using human iPSC-derived cortical neurons, forebrain organoids, and postmortem AD brain tissue, we evaluated J8 target engagement and its association with fibril-induced apoptotic and cellular-stress signals during concurrent exposure. These findings provide a human translational framework for evaluating Fc-free, conformation-selective anti-amyloid leads.

## Results

### Phage display identifies J8 as a fibril-selective, monomer-sparing anti-Aβ single-domain antibody

To isolate single-domain antibodies that selectively recognize pathogenic amyloid assemblies, we performed phage display screening against immobilized Aβ40 fibrils representing a patient-relevant fibril polymorph reported in AD (Figure 1A).^14^^,^^15^ Repeated rounds of panning progressively enriched phage populations for fibril binding. Polyclonal phage supernatants from rounds 2 and 3 showed significantly stronger binding to Aβ40 fibrils compared with round 1, while binding to Aβ40 monomer remained low, consistent with selective enrichment of fibril-binding clones (Figure 1B).

**Figure 1:**
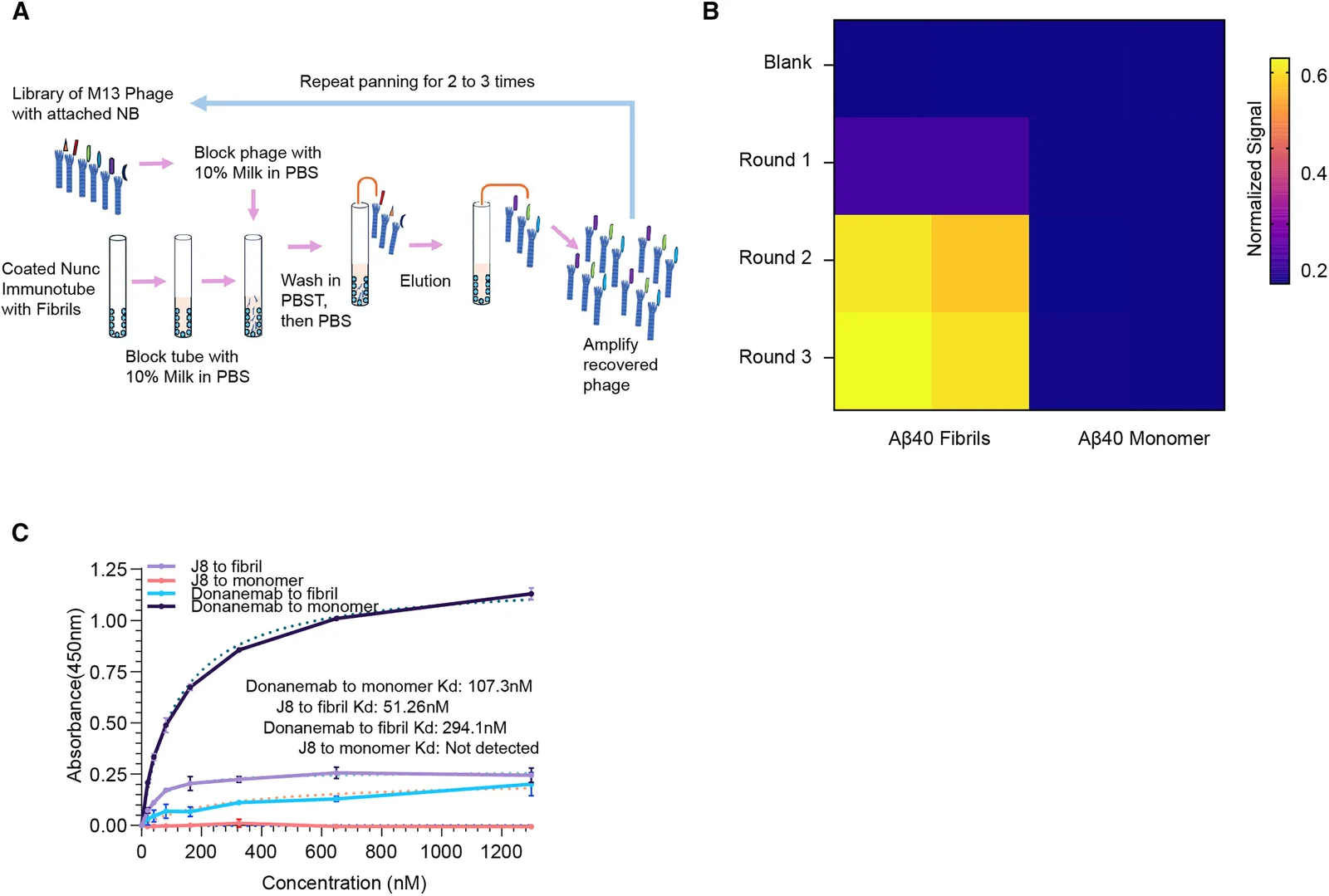
Selection and characterization of a fibril-selective anti-amyloid-β single-domain antibody (A) Schematic of the phage display screening workflow used to isolate single-domain antibodies that bind amyloid-β 40 fibrils. (B) Heatmap of ELISA binding of polyclonal phage supernatants from successive panning rounds to Aβ40 fibrils and Aβ40 monomer. (C) ELISA binding of single-domain antibody J8 and the comparator antibody donanemab to Aβ40 fibrils and Aβ40 monomer, with absorbance at 450 nm plotted as a function of antibody concentration. Data are mean ± SEM; *n* = 3. See also Figures S1–S3.

From this screen, we identified and purified the single-domain antibody J8 and its Avi-tagged counterpart, J8-Avi, for further characterization (Figures S1–S3). ELISA analysis showed that J8 bound Aβ40 fibrils with high apparent affinity (apparent Kd = 51.26 nM), whereas no detectable binding to Aβ40 monomer was observed under the same assay conditions (Figure 1C). In contrast, the control antibody donanemab bound both Aβ40 fibrils (apparent Kd = 294.1 nM) and Aβ40 monomer (apparent Kd = 107.3 nM), consistent with broader Aβ reactivity in this assay format (Figure 1C). The sequence of J8 and associated characterization data are provided as a resource (see method) to facilitate further investigation of fibril-selective Aβ targeting and drug development.

### J8 directly decorates Aβ fibrils and attenuates cleaved caspase-3 signaling during concurrent fibril exposure in human cortical neurons

We next tested whether J8 directly associates with Aβ40 fibrils using immunogold transmission electron microscopy. Immunogold transmission electron microscopy showed no gold particle labeling on fibrils incubated with PBS (Figure 2A). In contrast, fibrils incubated with J8 showed gold particles distributed along the fibril surface, confirming direct association of J8 with Aβ40 fibrils (Figure 2B).

**Figure 2:**
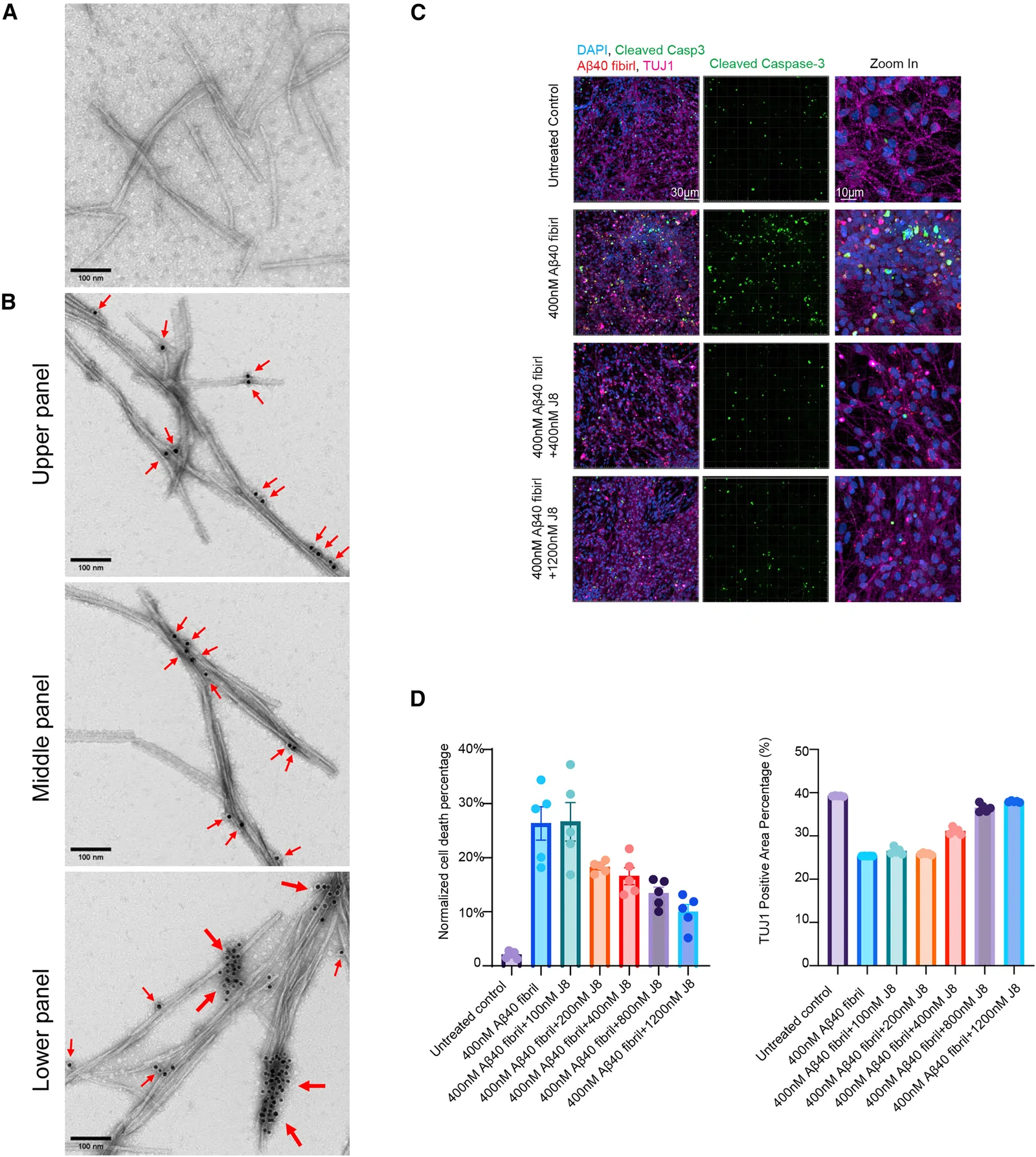
J8 binds Aβ40 fibrils and attenuates fibril-induced apoptotic signaling and morphological changes in human cortical neurons during concurrent exposure (A and B) Immunogold transmission electron microscopy showing binding of J8 to Aβ40 fibrils. Fibrils incubated with PBS show no gold-particle labeling (A), whereas fibrils incubated with J8-Avi-colloidal gold show gold particles along the fibril surface (B, red arrows; upper, middle, and lower images). Scale bars, 100 nm. (C) Representative immunofluorescence images of human iPSC-derived cortical neurons treated with Aβ40 fibrils in the presence or absence of increasing concentrations of J8, stained for TUJ1, cleaved caspase-3, and DAPI. Zoomed-in images highlight neuronal morphology and apoptotic-marker staining. (D) Quantification of cleaved caspase-3-positive signal following treatment with 400 nM Aβ40 fibrils in the presence of increasing concentrations of J8 (100–1,200 nM) (left) and quantification of TUJ1-positive area from the corresponding cultures (right). Data are mean ± SEM from five independent biological replicates.

To determine whether J8 attenuates fibril-associated neuronal injury during Aβ40 fibril exposure, human iPSC-derived cortical neurons were treated concurrently with Aβ40 fibrils and increasing concentrations of J8. Exposure to Aβ40 fibrils increased cleaved caspase-3 immunoreactivity, whereas concurrent J8 treatment resulted in a concentration-dependent reduction in cleaved caspase-3 signal (Figures 2C and 2D, left panel). Quantification of TUJ1-positive area across the treatment groups demonstrated a dose-dependent increase with increasing J8 concentration, consistent with partial preservation of neuronal morphology following Aβ40 fibril exposure (Figure 2D, right panel). Together, these findings indicate that concurrent J8 treatment attenuates Aβ40 fibril-associated apoptotic signaling and morphological alterations in human cortical neurons.

### J8 engages plaque-associated amyloid in human AD tissue and attenuates cleaved caspase-3 signaling during concurrent fibril exposure in forebrain organoids

We next tested whether J8 recognizes pathological amyloid assemblies in human AD tissue. Immunofluorescence staining of human brain tissue sections from a cognitively normal control with mild Aβ deposition showed weak and limited signal for both the pan-Aβ antibody 6E10 and J8. In contrast, AD brain sections showed strong plaque-like Aβ structures labeled by 6E10, and J8 co-localized with these amyloid deposits, indicating that J8 recognizes plaque-associated Aβ deposits in human AD tissue (Figure 3A).

**Figure 3:**
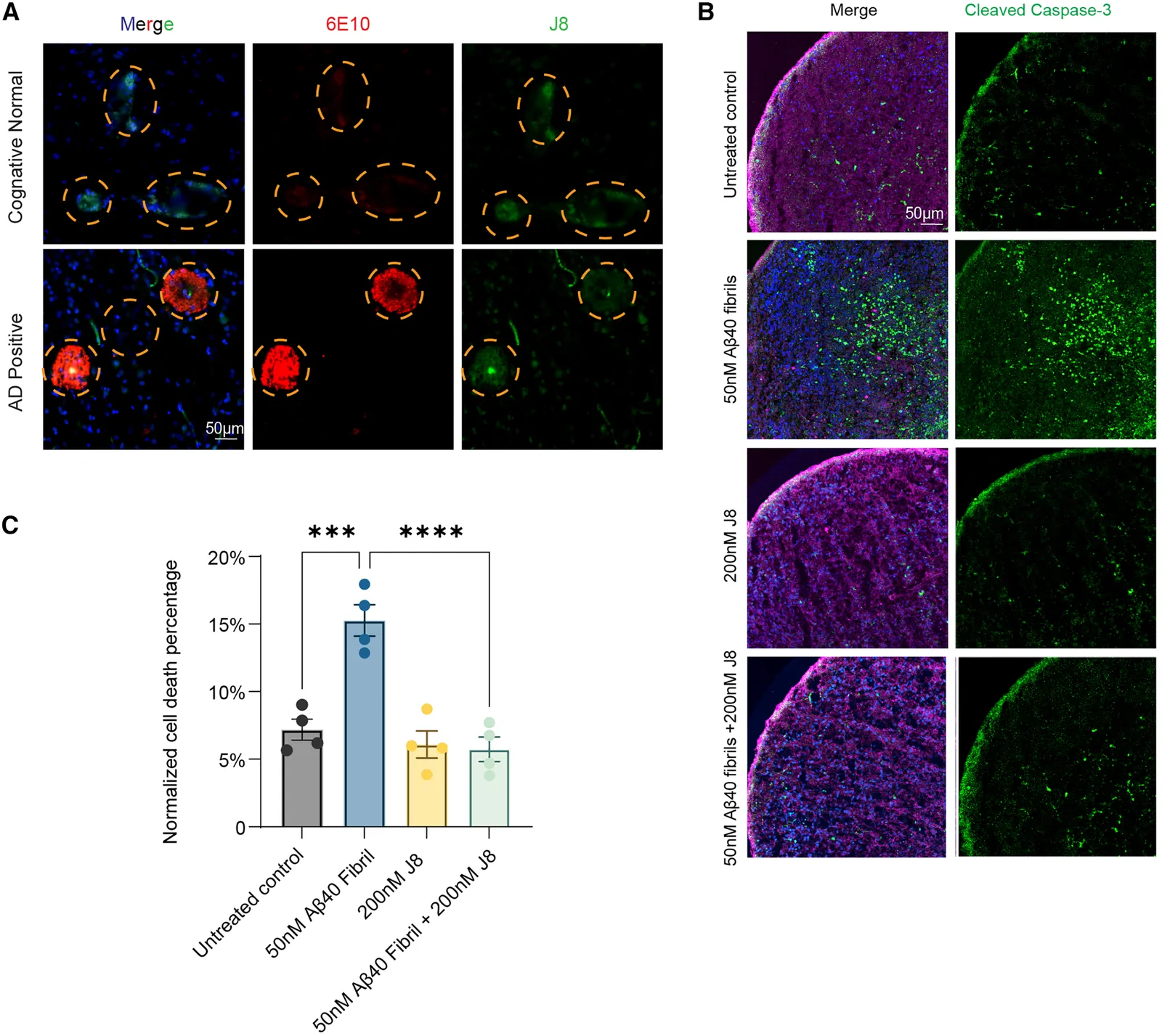
J8 recognizes amyloid pathology in human Alzheimer disease tissue and attenuates fibril-induced apoptotic signaling in a 3D cortical organoid model (A) Immunofluorescence staining of human brain sections from a cognitively normal control with mild Aβ deposition and from an Alzheimer disease patient, co-stained with the pan-Aβ antibody 6E10 and nanobody J8. Dashed circles indicate representative amyloid-positive regions. Scale bars, 50 μm. (B) Representative immunofluorescence images of 3D human cortical organoid slices treated with Aβ40 fibrils in the presence or absence of J8, stained for TUJ1, cleaved caspase-3, and DAPI. Scale bars, 50 μm. (C) Quantification of cleaved caspase-3-positive signal in untreated control, J8-alone, Aβ40 fibril-alone, and concurrent Aβ40 fibril-plus-J8 conditions. Data are mean ± SEM. ∗∗∗*p* < 0.001 and ∗∗∗∗*p* < 0.0001 by one-way ANOVA; *n* = 4 randomly selected organoids per condition from a pooled organoid set derived from three healthy control iPSC lines.

We next used human forebrain organoids to determine whether J8 attenuates cellular responses when present during Aβ40 fibril exposure. To this end, we differentiated two batches of forebrain organoids from three healthy control stem cell lines and treated organoids for 32 days under the indicated conditions. Organoids were assigned to untreated control, J8 alone, Aβ40 fibrils alone, or concurrent Aβ40 fibril plus J8 treatment. Aβ40 fibril exposure increased cleaved caspase-3 immunofluorescence signal, whereas concurrent treatment with J8 was associated with lower cleaved caspase-3 signal than fibril treatment alone (Figures 3B and 3C). The J8-alone condition did not show an increase in cleaved caspase-3 signal under the tested conditions. These findings demonstrate attenuation of fibril-associated apoptotic signaling during co-exposure but do not establish reversal of an established phenotype. Together, these results show that J8 recognizes plaque-associated amyloid and is associated with reduced cleaved caspase-3 signaling during concurrent Aβ40 fibril exposure in human forebrain organoids.

### Single-nucleus transcriptomic analysis identifies cellular-state changes associated with concurrent J8 treatment

We next used single-nucleus RNA sequencing (RNA-seq) to study how J8 alters the response of human forebrain organoids to Aβ fibrils. We analyzed PBS-treated control organoids, Aβ fibril-treated organoids, and organoids treated with fibrils together with J8. Stringent quality control and preprocessing were applied, including filtering of low-quality cells, removal of ribosomal-gene-dominated transcriptomes (Figure S4), ensuring robust identification of cell populations and downstream transcriptional changes. This analysis resolved five major cell populations, including excitatory neurons, inhibitory neurons, astrocytes, oligodendrocyte precursor cells, and perivascular cells, on the basis of canonical marker expression (Figures 4A and 4B; Table S1). In astrocytes, fibril treatment was associated with reduced expression of astrocyte-associated genes, including PTPRZ1, GFAP, and SLC1A2 (Figure 4C left panel), and increased expression of ER stress genes DDIT3, XBP1, and ATF4 (Figure 4D, left panel). During concurrent treatment, these expression changes were shifted in the opposite direction relative to fibril treatment alone and increased PTN expression, resulting in expression estimates close to those observed in the control condition (Figure 4C, left panel). In excitatory neurons, fibril treatment reduced expression of components of the NTN1-DCC/UNC5 signaling axis and lowered expression of synaptic and neuronal function genes, including CAMK2A, SNAP25, and DLG4, while increasing apoptosis-associated transcripts. These neuronal expression changes were less pronounced in the concurrent fibril-plus-J8 condition, although the transcriptional state remained distinct from control (Figures 4C and 4D, middle and right panels).

**Figure 4:**
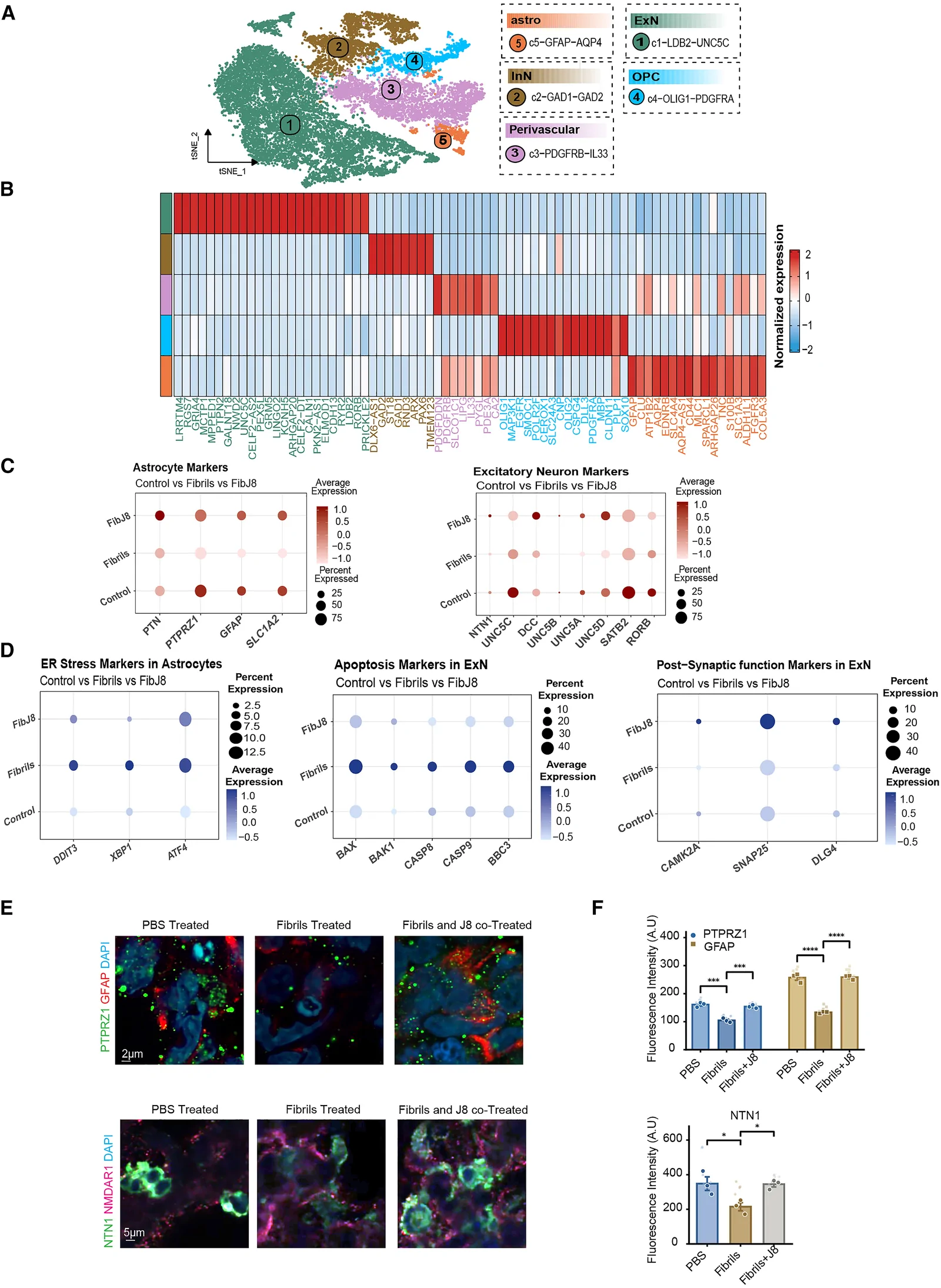
Single-nucleus transcriptomic analysis and immunostaining show that J8 reduces fibril-induced stress responses and partly restores astrocyte and excitatory neuron states in human forebrain organoids (A) t-SNE visualization of single-nucleus RNA sequencing data from control, fibril-treated, and fibril-plus-J8-treated organoids, resolving five major cell populations: excitatory neurons (ExN), inhibitory neurons (InN), astrocytes, oligodendrocyte precursor cells (OPC), and perivascular cells. (B) Heatmap of normalized expression of representative marker genes used for cluster annotation across the five populations; red indicates higher and blue lower expression. (C) Dot plots of selected genes associated with astrocyte PTN-PTPRZ1 signaling and excitatory neuron NTN1-UNC5 signaling across conditions. Dot size indicates the percentage of nuclei expressing each gene; color indicates average expression. (D) Dot plots of ER stress markers in astrocytes and apoptosis-related and post-synaptic function genes in excitatory neurons across conditions. (E) Representative immunofluorescence images of organoid sections showing PTPRZ1 (green), GFAP (red), and DAPI (blue) (top) and NTN1 (green), NMDAR1 (magenta), and DAPI (blue) (bottom) in PBS-treated, fibril-treated, and fibril-plus-J8-treated organoids. Scale bars as indicated. (F) Quantification of PTPRZ1 and GFAP fluorescence intensity (upper) and NTN1 fluorescence intensity in NMDAR1-positive cells (lower). Small circles represent individual cell measurements, large squares and rectangles represent per-organoid means, and bars indicate mean ± SEM of the three organoid-level means. Cell-level measurements were averaged within each organoid before statistical analysis, and only organoid-level means were treated as independent observations; *N* = 15 cells from n = 3 organoids per condition. ∗*p* < 0.05, ∗∗∗*p* < 0.001, and ∗∗∗∗*p* < 0.0001 by ordinary one-way ANOVA followed by Tukey’s multiple-comparisons test.

We next asked whether these transcriptional changes were also reflected at the protein level in organoids. Immunofluorescence staining showed that fibril treatment reduced PTPRZ1 and GFAP signals in astrocyte-enriched regions and reduced neuronal NTN1 in NMDAR1-positive excitatory neurons, consistent with the single-nucleus RNA-seq analysis. PTPRZ1, GFAP, and neuronal NTN1 signals were higher in the concurrent fibril-plus-J8 condition than in the fibril-only condition (Figure 4E). Quantification of fluorescence intensity confirmed significant reduction of PTPRZ1, GFAP, and NTN1 after fibril treatment, with higher signals observed during concurrent J8 treatment (Figure 4F). Organoid-level quantification showed lower mean PTPRZ1, GFAP, and NTN1 fluorescence in fibril-treated organoids than in PBS-treated controls and higher mean fluorescence in the concurrent fibril-plus-J8 condition than in the fibril-only condition (Figure 4F). These immunostaining results provide orthogonal protein-level support for the direction of change in selected markers but do not validate the broader transcriptome-wide pattern or overcome the absence of independently indexed sequencing replicates.

### Analysis identifies candidate J8-associated expression patterns in pooled human forebrain organoids

We restricted this analysis to nuclei annotated as excitatory neurons and compared fibril-treated versus control organoids (ΔF = log2CPM Fibrils−Control) and fibril-plus-J8-treated versus fibril-treated organoids (ΔJ = log2CPM FibJ8−Fibrils). To avoid treating individual nuclei as independent replicates, raw counts were aggregated within each condition-level library, and descriptive effects were computed on normalized values; cell-level Wilcoxon *p* values were not used for condition-associated inference. After restricting to protein-coding genes, applying effect-size thresholds (|ΔF| ≥ 0.10 and |ΔJ| ≥ 0.10), and requiring a mean expression of at least five CPM across conditions to limit unstable low-count log-ratios, 2,319 genes were retained for analysis.

A large subset showed opposite-direction effects across the two comparisons (Figure 5A). By quadrant, 1,343 genes were lower with fibrils and higher with J8 (Q2) and 561 showed the reverse (Q4), while few genes moved in the same direction across both comparisons (Q1, 209; Q3, 206) (Figure 5B). We refer to the Q2 genes as candidate genes showing opposite-direction effects. This asymmetry, in which fibril-associated changes tend to be countered by J8, is the central observation and is mirrored when genes are stratified by their fibril-effect direction (Figure 5C).

**Figure 5:**
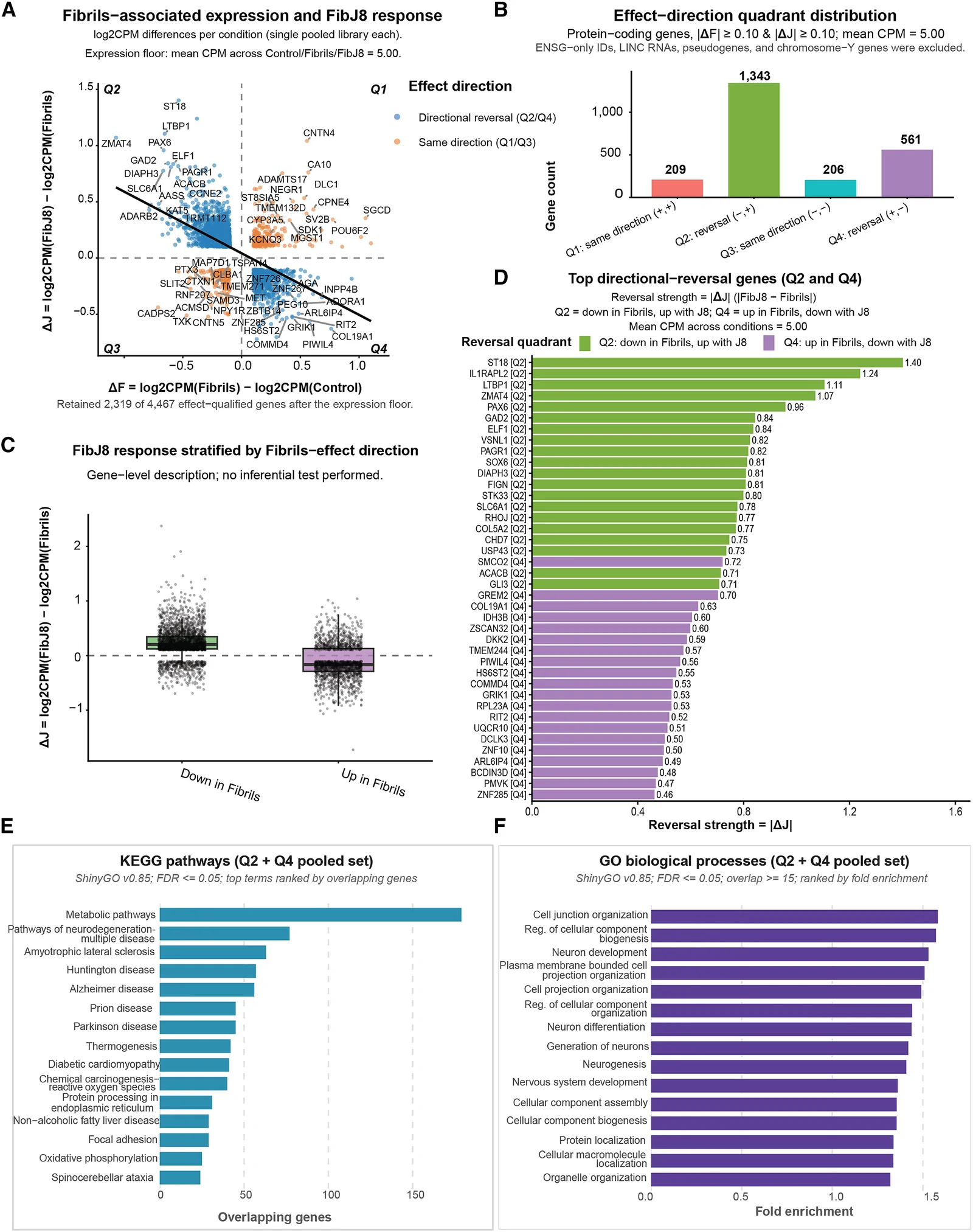
Candidate J8-associated directional-reversal expression patterns in excitatory neurons of human forebrain organoids (A) Scatterplot of per-gene effects, ΔF = log2CPM(Fibrils−Control) versus ΔJ = log2CPM(FibJ8−Fibrils), for protein-coding genes passing the expression floor (mean CPM ≥5). Genes with opposite-direction effects across the two comparisons (Q2/Q4) are shown in blue and same-direction genes (Q1/Q3) in orange; selected genes are labeled. (B) Quadrant distribution of effect-qualified genes (|ΔF| ≥ 0.10 and |ΔJ| ≥ 0.10): Q1, 209; Q2, 1,343; Q3, 206; Q4, 561. (C) ΔJ for genes stratified by the direction of their fibril-associated effect. This panel is descriptive, and no inferential test was performed. (D) Top directional-reversal candidates ranked by reversal strength (|ΔJ|), shown for Q2 (down in Fibrils, up with J8) and Q4 (up in Fibrils, down with J8). (E) KEGG pathways overrepresented among the pooled Q2+Q4 directional-reversal set (ShinyGO v0.85, false discovery rate [FDR] ≤0.05). (F) Gene Ontology biological process terms for the same gene set. All effects are descriptive summaries from single pooled libraries per condition.

Ranking candidate genes showing opposite-direction effects by strength (|ΔJ|), the top Q2 candidates—those suppressed by fibrils and higher in the FibJ8 library than in the fibril library—were dominated by neuronal and synaptic genes, including ST18, IL1RAPL2, LTBP1, ZMAT4, PAX6, GAD2, VSNL1, SOX6, and SLC6A1 (Figure 5D). Notably, VSNL1 (visinin-like protein 1), a neuronal calcium sensor whose expression is reduced in the AD brain in proportion to neurofibrillary pathology, appeared among the genes suppressed by fibrils and improved expression in the FibJ8 library, directionally consistent with the human disease.^29^^,^^30^ IL1RAPL2, which promotes excitatory synapse and dendritic spine formation, showed the same pattern.^31^ These examples suggest a set of candidates for future functional studies.

Additional analysis of the directional-reversal set recovered neurodegeneration and neuronal pathways. KEGG terms included pathways of neurodegeneration, amyotrophic lateral sclerosis, Huntington disease, AD, and Parkinson disease (Figure 5E), while Gene Ontology biological process terms centered on neuron development, differentiation, neurogenesis, and cell projection organization (Figure 5F; Table S2).

Because each condition was a single pooled library rather than independently indexed replicates, these estimates prioritize candidates rather than establish treatment effects, and no inferential testing was performed.

### Fibril-associated organoid changes move in the human Alzheimer disease direction and are opposed by J8

To test whether fibril-associated changes are disease-relevant, we compared the sign of our organoid Fibrils-versus-Control log2 fold changes against published excitatory-neuron summaries from human AD cortex: Leng et al. (entorhinal cortex: Braak 2 versus 0 and Braak 6 versus 0)^32^ and Morabito et al. (AD versus control).^33^ For genes shared with each dataset, we tested sign concordance against the 50% chance expectation with a two-sided binomial test. These independently replicated human datasets serve only as an external reference for the disease direction and do not assess the J8 response.

Concordance was stage-dependent (Figures 6A–6D; Table S2). The early-stage Leng comparison did not differ from chance (47.5%; *n* = 118; *p* ≈ 0.65), whereas the late-stage comparison was well above chance (60.3%; *n* = 358; *p* ≈ 1 × 10^−4^), as was Morabito more modestly (56.3%; *n* = 492; *p* ≈ 6 × 10^−3^). Above-chance agreement emerging only at advanced Braak stage is consistent with the fibril-treated state resembling later-stage disease, though the sign-based test implies directional rather than quantitative correspondence.

**Figure 6:**
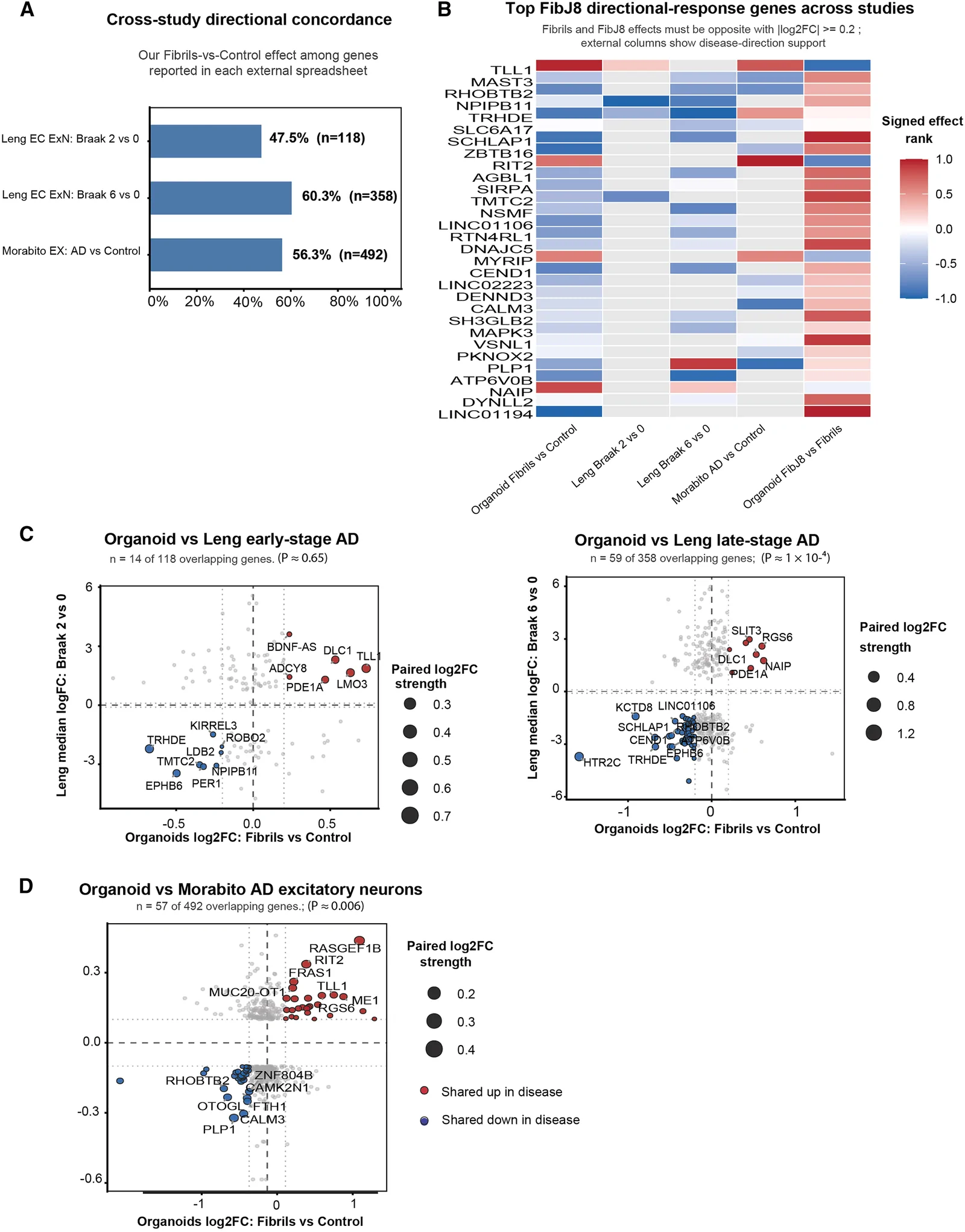
Fibril-associated organoid changes show stage-dependent concordance with human Alzheimer disease and are opposed by J8 (A) Directional concordance between the organoid Fibrils-versus-Control effect and each human AD dataset, shown as the percentage of shared genes with the same effect sign: Leng Braak 2 versus 0 (47.5%, *n* = 118), Braak 6 versus 0 (60.3%, *n* = 358), and Morabito AD versus control (56.3%, *n* = 492). (B) Signed-rank heatmap of top FibJ8 directional-response genes, required to show opposite Fibrils and FibJ8 effects (|log2FC| ≥ 0.2), ranked by external disease-direction support and then reversal strength, with sex-associated genes excluded. Values within each column are signed ranks (−1 to 1). Columns, left to right: organoid Fibrils effect, Leng Braak 2, Leng Braak 6, Morabito, and organoid FibJ8. (C) Organoid Fibrils-versus-Control log2 fold changes versus Leng early-stage (left) and late-stage (right) AD, with same-direction genes highlighted; point size reflects paired effect-size strength. (D) As in (C) for Morabito, with genes colored by shared-up or shared-down direction. *p* values are from two-sided binomial tests against a 50% null. All organoid estimates are descriptive (single pooled library per condition); external datasets support only the disease direction.

We then asked whether J8 opposes these changes. Ranking FibJ8 directional-reversal candidates (opposite Fibrils and FibJ8 effects, |log2FC| ≥ 0.2) by the number of external datasets supporting the disease direction, the FibJ8 column consistently opposed both the fibril and human-disease columns (Figure 6B): genes whose fibril-associated changes matched the AD direction tended to move oppositely with J8.

Concordance was more pronounced at the pathway level (Figure 7; Table S2). Preranked gene set enrichment analysis (GSEA) of the organoid and each disease vector, compared by normalized enrichment score (NES) sign, again showed a stage-dependent pattern: the early-stage Leng comparison had few overlapping pathways and little agreement (GO-BP 8.0%, 2 of 25; Reactome 50.0%, 1 of 2), whereas the late-stage comparison showed majority agreement (GO-BP 61.3%, 103 of 168; Reactome 69.6%, 16 of 23), with Morabito showing the strongest agreement (GO-BP 70.4%, 264 of 375; Reactome 87.9%, 29 of 33). Concordant pathways were predominantly downregulated in both datasets and centered on synaptic and neuronal processes, including synapse assembly, *trans*-synaptic signaling, and NMDA receptor and postsynaptic signaling, with a smaller set of shared upregulated terms such as glutamate receptor signaling.

**Figure 7:**
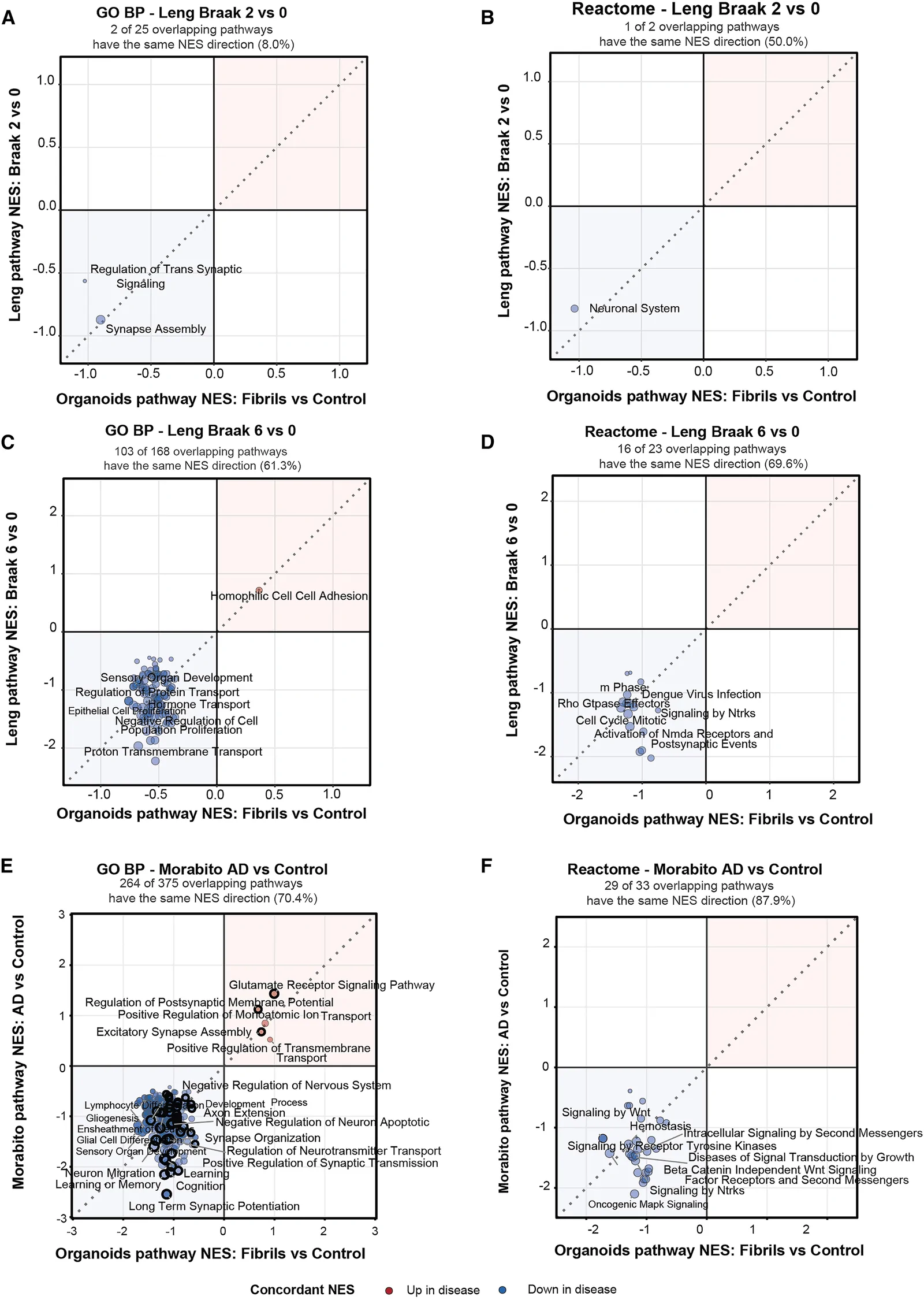
Pathway-level concordance between the fibril-associated organoid state and human Alzheimer disease (A and B) Preranked GSEA of the organoid Fibrils-versus-Control effect and the Leng Braak 2 versus 0 vector, comparing normalized enrichment scores for GO biological process (A) and Reactome (B) gene sets: 8.0% (2 of 25) and 50.0% (1 of 2) concordant, respectively. (C and D) As in (A) and (B) for Leng Braak 6 versus 0: 61.3% (103 of 168) and 69.6% (16 of 23). (E and F) As in (A) and (B) for Morabito AD versus control: 70.4% (264 of 375) and 87.9% (29 of 33). Each point is a shared pathway; pathways with matching NES sign are concordant and colored by whether they are up (red) or down (blue) in disease. Organoid estimates are descriptive (single pooled library per condition); external datasets support only the disease direction.

Together, a subset of fibril-perturbed genes moves in the human AD direction, most clearly at later disease stages and more robustly at the pathway level, and J8 is associated with opposing, disease-reversing changes in these genes.

## Discussion

In this study, we identified J8 as a fibril-selective single-domain antibody that recognizes Aβ assemblies modeled on a prevalent patient-derived fibril polymorph and mitigates fibril-associated injury in human neuronal systems. J8 bound Aβ40 fibrils with high apparent affinity and showed no detectable binding to monomeric Aβ40 in the same assay format, whereas the comparator antibody donanemab bound both fibrils and monomer. J8 directly associated with fibril surfaces by immunogold electron microscopy, recognized plaque-like amyloid deposits in AD brain tissue, and reduced fibril-induced apoptotic signaling in both human iPSC-derived cortical neurons and forebrain organoids. Single-nucleus transcriptomic analysis further indicated that J8 blunted fibril-induced stress programs and partly preserved astrocytic and excitatory neuronal states. Together, these findings support further characterization of J8 as a potential anti-amyloid lead. Although J8 preferentially recognized the tested Aβ40 fibril preparation relative to monomer, the optimal Aβ assembly state for therapeutic targeting remains unresolved. Soluble oligomers, protofibrils, and mature fibrils may contribute to AD pathology through distinct mechanisms, and reduction of fibrillar plaque burden has not consistently translated into clinical benefit. Conversely, clinically active anti-Aβ antibodies have different epitope and aggregate-binding profiles, complicating attribution of their effects to engagement of a single Aβ assembly state. Therefore, the present findings should not be interpreted as establishing mature fibrils as the preferred therapeutic target. Comparative evaluation of J8 against oligomeric, protofibrillar, and structurally distinct fibrillar Aβ preparations will be required to define its broader selectivity and therapeutic relevance.

The absence of an Fc domain represents a potential therapeutic trade-off. An Fc-free binder may reduce Fc receptor- and complement-mediated inflammatory signaling; however, Fc-dependent engagement of microglia may also contribute to the uptake and clearance of antibody-bound Aβ deposits. Removal of the Fc domain could therefore reduce plaque-clearance activity and compromise therapeutic efficacy. The present neuronal and organoid models do not contain a physiologically complete immune or vascular compartment and cannot resolve the balance between reduced inflammatory signaling and reduced Fc-dependent clearance. Accordingly, the Fc-free format of J8 should be regarded as a design hypothesis rather than an established therapeutic advantage, and its efficacy and safety will require direct evaluation in appropriate *in vivo* models.

The data support that J8 acts directly at the level of fibrillar Aβ. The immunogold electron microscopy data demonstrate physical association of J8 with fibril surfaces *in vitro*. At present, however, the mechanism remains unresolved. The current data do not distinguish whether J8 caps fibril ends, masks toxic fibril surfaces, suppresses secondary nucleation, or remodels fibrils into less bioactive assemblies. Future structural and biophysical studies will therefore be important to map the J8 epitope and define the specific aggregation step affected by binding.

The neuronal and organoid experiments were designed to evaluate the effect of J8 during concurrent exposure to Aβ40 fibrils. In both models, J8 co-treatment was associated with reduced cleaved caspase-3 signaling, and in cortical neurons, a dose-dependent increase in TUJ1-positive area, supporting attenuation of fibril-associated apoptotic signaling and preservation of neuronal morphology during co-exposure. However, these experiments were not designed to determine whether J8 can reverse an established cellular phenotype after fibril-induced injury has developed. Moreover, although ELISA and immunogold electron microscopy independently demonstrate binding of J8 to the tested Aβ40 fibrils, the cellular studies did not include a matched non-targeting single-domain antibody or a J8-alone control in the 2D neuronal assay. Accordingly, the present findings support attenuation of fibril-associated responses during concurrent treatment but do not establish therapeutic rescue, target-specific neuroprotection, or efficacy after pathology has been initiated. These questions will require delayed-addition studies, appropriate control antibodies, and complementary assessments of neuronal viability and function.

The single-nucleus transcriptomic data provide additional insight into the cellular programs altered by fibril exposure and modulated by J8. In astrocytes, fibril treatment was associated with suppression of astrocyte-associated genes and induction of ER stress-related transcripts, whereas J8 partly attenuated these changes. In excitatory neurons, fibril exposure reduced expression of genes linked to neuronal connectivity and synaptic function and increased apoptosis-associated programs, and these alterations were also attenuated by J8. Recovery of selected astrocytic and neuronal protein markers in organoids supports the biological relevance of these transcriptional findings. Importantly, the J8-associated shift toward the control state was partial rather than complete, and the treated organoids did not fully return to the untreated state. This pattern is biologically plausible and suggests that neutralizing fibril-associated toxicity can alleviate downstream stress responses without immediately normalizing all injury-associated programs.

To assess whether these fibril-associated transcriptional changes are relevant to human disease, we compared them against independently generated single-nucleus datasets from postmortem AD cortex. The direction of the fibril-associated organoid changes agreed with human AD in a stage-dependent manner, showing little correspondence at early Braak stage but concordance exceeding chance at late Braak stage and in an independent AD cohort, with stronger agreement at the level of pathways than individual genes. The predominance of shared downregulation of synaptic and neuronal programs is consistent with the loss of synaptic and neuronal identity that characterizes AD cortex. Because these external datasets derive from replicated human cohorts, this convergence provides independent support that the organoid signature reflects disease-relevant biology rather than a technical artifact of the pooled design. At the same time, the correspondence was directional rather than quantitative and was clearest at advanced pathology, suggesting that acute fibril exposure in organoids models features of later-stage disease and does not reproduce the full transcriptional architecture of human AD. Notably, among these disease-concordant genes, the J8 condition was associated with changes in the opposing direction, nominating a prioritized, externally supported set of candidate genes; however, because the external data speak only to the disease direction and the organoid estimates are descriptive, the J8-associated opposite-direction pattern remains a hypothesis for validation in replicated experiments. Because each condition was represented by a single pooled library, all condition-level transcriptomic comparisons (Figures 5, 6, and 7) are descriptive; the human AD datasets supply independently replicated support only for the disease direction of the fibril effect, and confirming the J8-associated opposite-direction pattern will require replicated organoid experiments analyzed with replicate-aware methods.

Another important finding is that J8, which was selected against synthetic Aβ40 fibrils designed to resemble a patient-relevant polymorph, also recognized plaque-like amyloid deposits in AD brain tissue. This result strengthens the translational significance of the J8 by showing that the selected epitope is not limited to the *in vitro* immunogen. At the same time, human amyloid pathology is heterogeneous across patients, brain regions, and plaque versus vascular deposits. It will therefore be important to test J8 across a larger panel of postmortem AD tissues, determine its reactivity toward additional Aβ conformers and polymorphs, and assess whether it shows differential recognition of parenchymal plaques and cerebral-amyloid-angiopathy-associated deposits.

An additional implication of this study is methodological. The combination of human forebrain organoids and single-nucleus transcriptomics provides a human-relevant, fit-for-purpose framework for preclinical evaluation of conformation-selective anti-amyloid biologics. Recent FDA draft guidance on NAMs in drug development emphasizes clear context of use, human biological relevance, technical characterization, and interpretation within a weight-of-evidence framework. In that context, the organoid platform used here is best viewed not as a replacement for *in vivo* studies but as a multicellular human system that fills a preclinical data gap between biochemical target-engagement assays and later-stage animal or translational studies by enabling direct assessment of fibril-associated cellular injury and state changes.

Beyond its potential therapeutic relevance, J8 and the associated organoid/single-nucleus workflow provide a human-relevant resource for interrogating pathogenic Aβ assemblies and evaluating conformation-selective anti-amyloid strategies in multicellular neural systems. The availability of its sequence, together with associated transcriptomic datasets, may facilitate studies of amyloid biology, target engagement, and conformation-selective therapeutic strategies.

Together, these findings establish J8 as a fibril-selective, monomer-sparing anti-Aβ nanobody that engages pathological amyloid in human-relevant systems and supports further development of Fc-free, conformation-selective approaches for targeting Aβ in AD.

### Limitations of the study

Several limitations should be considered when interpreting these findings. J8 binding was evaluated against a limited set of Aβ preparations; therefore, its epitope, breadth of conformer recognition, and selectivity across distinct fibril polymorphs remain to be established. Likewise, relative sparing of monomeric Aβ was demonstrated only under the assay conditions used here and should not be generalized to other Aβ species or experimental contexts. The neuronal experiments used concurrent exposure to Aβ40 fibrils and J8 and therefore demonstrate attenuation of selected fibril-associated responses during co-treatment rather than reversal of established pathology or therapeutic efficacy. The 2D neuronal cultures and forebrain organoids do not fully recapitulate the microglial, vascular, and systemic components of AD, and the human tissue analysis was limited to postmortem samples from two donors with AD and two cognitively normal donors. In addition, because the single-nucleus RNA-seq experiment used one pooled library per condition, the condition-level transcriptomic comparisons are descriptive and hypothesis-generating and do not provide biological-replicate-level inference. The study also did not directly compare Fc-free and Fc-containing J8 formats or assess *in vivo* pharmacokinetics, target engagement, efficacy, immunogenicity, amyloid clearance, or ARIA-related safety. Future studies should therefore define the J8 epitope and conformer selectivity, optimize affinity and developability, test delayed-treatment paradigms and independently replicated human models incorporating microglial and vascular components, and evaluate efficacy and safety *in vivo*.

## Resource availability

### Lead contact

Requests for further information and resources and reagents should be directed to and will be fulfilled by the lead contact and corresponding author, Dr. Qi Zhang (qi.zhang3@nih.gov).

### Materials availability

The J8 (NCATS-BL0252) and J8-Avi (NCATS-BL0253) single-domain antibodies generated in this study are available from Bryan Fleming (bryan.fleming@nih.gov) or Qi Zhang (lead contact) upon request and completion of an NIH material transfer agreement (MTA). The amino acid sequences of J8 and J8-Avi are provided below.

#### >J8

EVQLVESGGGLVQPGGSLRLSCAASGSISSKRMGWFRQAPGKGRELVASIGYGSNTYYPDSVEGRFTISRDNAKRMVYLQMNSLRAEDTAVYYCAVADYRTYVDSFDWGQGTQVTVSSGQAGQHHHHHHGAYPYDVPDYAS∗

#### >J8-Avi

EVQLVESGGGLVQPGGSLRLSCAASGSISSKRMGWFRQAPGKGRELVASIGYGSNTYYPDSVEGRFTISRDNAKRMVYLQMNSLRAEDTAVYYCAVADYRTYVDSFDWGQGTQVTVSSGQAGQHHHHHHGAYPYDVPDYASGLNDIFEAQKIEWHE∗

### Data and code availability

• Single-nucleus RNA-seq data have been deposited at GEO and are publicly available as of the date of publication under accession number GSE325804. The accession number is listed in the key resources table.
• All original code has been deposited at Zenodo at https://doi.org/10.5281/zenodo.22661968 and is publicly available as of the date of publication.
• Any additional information required to reanalyze the data reported in this paper is available from the lead contact upon request.

## Acknowledgments

We would like to thank NIDDK advanced imaging core for assistance of data acquisition and Dr. Yihong Ye of NIDDK for valuable inputs. This work is supported by the Intramural Research Programs of the National Institute of Diabetes and Digestive and Kidney Diseases (NIDDK) and the National Center for Advancing Translational Sciences (NCATS) within the National Institutes of Health (NIH). The contributions of the NIH authors are considered Works of the United States Government. The findings and conclusions presented in this paper are those of the authors and do not necessarily reflect the views of the NIH or the U.S. Department of Health and Human Services. This work is also supported by grants from NIH/NIA U19AG068054 (E.H.), NIH/NIA P30AG066519 (E.H.), NIH/NIA T32AG073088 (E.H.).

## Author contributions

M.A.G., S.A., B.D.F., R.T., and Q.Z. performed experiments and analyzed data. I.R., J.S., B.D.F., and N.J.M. contributed to experimental design and execution. A.S. and R.T. provided conceptual input and contributed to study design. E.H. designed human tissue studies. B.D.F. and Q.Z. supervised the study. Q.Z. conceived the project. M.A.G., S.A., B.D.F., and Q.Z. wrote the manuscript with input from all authors.

## Declaration of interests

The authors declare no competing interests.

## Declaration of generative AI and AI-assisted technologies in the manuscript preparation process

During the preparation of this work, the author(s) used [ChatGPT] in order to [assist language editing]. After using this tool or service, the author(s) reviewed and edited the content as needed and take(s) full responsibility for the content of the publication.

## STAR★Methods

### Key resources table

**Table:** 

REAGENT or RESOURCE	SOURCE	IDENTIFIER
**Antibodies**		

Goat Polyclonal anti-Human IgG (H + L)-HRP Antibody	Thermo Fisher Scientific	Cat#31410; RRID: AB_228269
Mouse Monoclonal Anti-M13-HRP Antibody	SinoBiological	Cat#11973-MM05T-H;RRID: AB_2857928
Mouse Monoclonal Anti-HA-HRP Antibody	GenScript	Cat#A01296; RRID: AB_1575850
Rabbit NTN1 polyclonal antibody	Proteintech	Cat#20235-1-AP; RRID: AB_2918065
Rabbit PTPRZ1 polyclonal antibody	Proteintech	Cat#55125-1-AP; RRID: AB_10858795
Mouse Anti-amyloid(6E10) antibody	Biolegend	Cat#803014; RRID: AB_2728527
Rabbit cleaved caspase-3 antibody	Cell signaling	Cat#99661; RRID:mAB_2341188
Mouse monoclonal anti-TUJ1	GeneTex	Cat#GTX631836; RRID: AB_2814952
Donanemab	Medchem express	Cat#1931944–80-7
J8 single-domain antibody (NCATS-J8)	This paper	NCATS-BL0252
J8-Avi single-domain antibody (NCATS-J8-Avi)	This paper	NCATS-BL0253

**Bacterial and virus strains**		

TG1 DUOs Electrocompetent *Escherichia coli*	Biosearch Technologies	Cat#60502–2
HB2151 *Escherichia coli*	Lifescience market	Cat#S0122
M13K07 Helper Phage	Cytiva	Cat#27–1524-01

**Biological samples**		

t-AD6f brain-derived Aβ40 fibril seed	Qiang et al.^14^/Ghosh et al.^15^	N/A
Postmortem Alzheimer’s disease brain tissue	University of California, Irvine	N/A
Cognitively normal control brain tissue	University of California, Irvine	N/A

**Chemicals, peptides, and recombinant proteins**		

Alexa Fluor™ 488 Streptavidin, 10 nm colloidal gold conjugate	Thermo Fisher Scientific	Cat#A32361
Triethylamine	Sigma-Aldrich	Cat#471283-100 ML
SuperBlock Blocking Buffer	Thermo Fisher Scientific	Cat#37515

**Critical commercial assays**		

Enzymatic Protein Biotinylation Kit	Sigma-Aldrich	Cat#CS0008

**Deposited data**		

Single-nucleus RNA-seq data	This paper	GEO: GSE325804
Single-Nucleus RNA sequencing Computational Workflow for Alzheimer Disease.	This paper	https://doi.org/10.5281/zenodo.22661968

**Experimental models: Cell lines**		

Human healthy-control iPSC panel, comprising lines (DXG-FB01-01, DXG-FB01-02,DXG-FB01-03)	Dexorgen, Inc	DXG-FB01

**Oligonucleotides**		

BF-Pr68: TAACAAAGCCCGAAAGGAAGCTG	This paper	N/A
BF-Pr69: CATGGTATATCTCCTTCTTAAAGTTA AACAAAATTATTTCTAG	This paper	N/A
BF-Pr70: AAGAAGACAGCTATCGCGATTGC	This paper	N/A
BF-Pr71: CAGCTTCCTTTCGGGCTTTGTTACTCATGC CACTCGATCTTCTGGGCCTCAAAGATGTC ATTCAGGCCAGAAGCGTAGTCCGGAACGT	This paper	N/A

**Recombinant DNA**		

pComb3XLambda NCATS-J8(NCATS-BL0252)	This paper	NCATS-J8
pET-28a(+) NCATS-J8-Avi (NCATS-BL0253)	This paper	eBF5

**Software and algorithms**		

R version 4.5.2	R Core Team/R Foundation for Statistical Computing	https://www.r-project.org/
Seurat v5	Hao et al.^34^	https://satijalab.org/seurat/
scDblFinder	Germain et al.^35^	https://bioconductor.org/packages/scDblFinder/
SCTransform (glmGamPoi method)	Ahlmann-Eltze & Huber^36^	https://bioconductor.org/packages/glmGamPoi/
metap	Dewey^37^	https://cran.r-project.org/package=metap
fgsea	Korotkevich et al.^38^	https://bioconductor.org/packages/fgsea/
msigdbr	Dolgalev^39^	https://cran.r-project.org/package=msigdbr
ShinyGO v0.85	Ge et al.^40^	http://bioinformatics.sdstate.edu/go/
Cell Ranger v7.0	10× Genomics^41^	https://www.10xgenomics.com/support/software/cell-ranger
GraphPad Prism v9.0	GraphPad Software^42^	https://www.graphpad.com/
Fiji/ImageJ	Schindelin et al.^43^	https://imagej.net/software/fiji/
Imaris	Oxford Instruments (Bitplane)	https://imaris.oxinst.com/
ComplexHeatmap	Gu et al.^44^	https://bioconductor.org/packages/ComplexHeatmap/
SingleR	Aran et al.^45^	https://bioconductor.org/packages/SingleR/

### Experimental model and study participant details

#### Human iPSC lines

Three independent healthy-control human induced pluripotent stem cell (iPSC) lines (DXG-FB01-01, DXG-FB01-02, and DXG-FB01-03; Dexorgen, Inc.) were used for cortical neuron and forebrain organoid differentiation. The panel comprised one iPSC line derived from a male donor and two iPSC lines derived from female donors. The cell lines were not independently authenticated in this study. Mycoplasma contamination was monitored by regular testing, and all lines tested negative throughout the study. The iPSCs were maintained on Matrigel-coated culture vessels under the conditions described below and were differentiated into cortical neurons and forebrain organoids as described in the corresponding method details sections. Information regarding donor age, ancestry, race, ethnicity, and genotype was not available to the investigators. Because the study included only three healthy-control iPSC lines and was not designed or powered to evaluate sex-associated differences, the influence of donor sex on the experimental outcomes could not be determined.

#### Postmortem human brain tissue

Postmortem human brain tissue was obtained from the University of California, Irvine Alzheimer’s Disease Research Center (UCI ADRC) Brain Repository. Tissue from two donors with neuropathologically confirmed Alzheimer’s disease and two cognitively normal donors was used in this study. Brain donation, tissue collection, and repository distribution were conducted under a protocol approved by the UCI Office of Research Human Research Protections (IRB protocol no. 20141526). Written informed consent for brain donation was obtained from donors during life and/or authorization for postmortem donation and research use was obtained from legally authorized next of kin, in accordance with UCI ADRC procedures. All specimens were provided to the investigators in de-identified form.

#### Bacterial strains

*E. coli* TG1 cells were used for phage reinfection and phage production. *E. coli* HB2151 cells were used for single-domain antibody expression.

### Method details

#### Generation of fibrils

Aβ40 fibrils for all experiments, except TEM measurements in Figure 2, were grown by seeding with Aβ40 fibrils that had been seeded with amyloid from human AD brain tissue as previously described.^14^^,^^15^ Briefly, recombinant Aβ40 (rPeptide) was fully dissolved in dimethyl sulfoxide at 7.5 mM Aβ40 concentration, then diluted to 200 μM in 0.9 mL of 10 mM sodium phosphate buffer, pH 7.4, with 0.01% w/v sodium azide. The buffer contained sonicated brain-derived Aβ40 fibrils (t-AD6f fibril sample from reference^14^^,^^15^) at approximately 10 μM Aβ40 concentration, which served as the fibril seeds. After incubation for 6 days under quiescent conditions at 24°C, seeded fibril growth was confirmed by TEM. For TEM measurements in Figure 2, Aβ40 fibrils were grown in the same way, but using sonicated synthetic fibrils with a “striated ribbon” morphology^46^ as fibril seeds, with an 8 μM Aβ40 concentration for the seeds.

#### Phage panning

Phage panning was performed using NCATS’ synthetic humanized nanobody library 2.0 according to a previously published protocol.^47^ Briefly, Aβ40 fibrils provided by the Tycko lab were sonicated in a water bath for 1 min, then 5 μg were diluted in 500 μL PBS. Sample was added to a Maxisorp immunotube overnight at 4°C with rocking. Coating solution was replaced with blocked phage and incubated for 1.5 h. Tubes were washed with 20 mL of PBS/Tween (0.05%), followed by 20 mL of PBS. Phage was eluted by adding 500 μL of Triethylamine for 15 min at room temperature. Recovered phage solution was neutralized using 250 μL of Tris-HCl pH 7.4, then added to TG1 cells for reinfection and subsequent phage productions steps. Panning was performed a total of four rounds to allow for phage enrichment.

#### Polyclonal phage ELISA

Polyclonal phage produced after each round of panning was tested by ELISA for binding activity. Nunc Maxisorp ELISA plates were coated with 50 μL Aβ40 fibrils or Aβ40 monomer at 5 μg/mL per well in PBS. Plates were incubated overnight at 4°C with rocking. Clarified phage was mixed 1:1 with ELISA phage block (3% milk in Superblock) for 1 h at room temperature. Plates were blocked in the same fashion after coating solution was removed. Plates had blocking solution removed and were replaced with the 100 μL blocked phage. Wells were incubated for 1 h with gentle rocking at room temperature. Plates were washed three times with ELISA wash buffer (PBS/Tween (0.05%)), then wells had 50 μL secondary 1:5000 diluted anti-M13-HRP prepared in ELISA phage block. Wells were incubated for 1 h with rocking, then wells were washed 6 times with ELISA wash buffer. Each well had 50 μL of TMB substrate added for 10 min, then reaction was stopped with 50 μL of 0.2 M H_2_SO_4_. The absorbance (450 nm) was determined on a Pherastar plate reader.

#### Plasmid construction

J8-Avi was generated by PCR amplifying the J8 nanobody coding sequence from NCATS-J8 plasmid (BL0252) using the forward primer BF-Pr70 (AAGAAGACAGCTATCGCGATTGC) and the Avi tag containing reverse primer BF-Pr71 (CAGCTTCCTTTCGGGCTTTGTTACTCATGCCACTCGATCTTCTGGGCCTCAAAGATGTCATTCAGGCCAGAAGCGTAGTCCGGAACGT). Backbone DNA for the construct was PCR amplified from the pET28a(+) vector using primers BF-Pr68 (TAACAAAGCCCGAAAGGAAGCTG) and BF-Pr69 (CATGGTATATCTCCTTCTTAAAGTTAAACAAAATTATTTCTAG). J8-Avi was fused into backbone DNA using a third PCR reaction using primers BF-Pr69 and BF-Pr70. Prior to transformation into TG1 cells, linear DNA was phosphorylated and ligated using T4 Polynucleotide Kinase and T4 DNA Ligase.

#### Protein production

HB2151 chemically competent cells (25 μL) were transformed with 300 ng of J8 or J8-Avi plasmid using 1 min heat shock at 42°C. Cells were chilled on ice for 2 min before adding 200 μL of SOC media. Transformations were then incubated at 37°C with shaking for 1 h. Cells were spread on 2XYT plates supplemented with 2% glucose and 100 μg/mL ampicillin and grown at room temperature for 3 days. Transformed cells were resuspended and transferred to 50 mL of 2XYT media supplemented with 2% glucose and 100 μg/mL ampicillin to reach a starting OD600 of 0.0025. Following 4 h of growth at 37°C with shaking, cells were collected by centrifugation for 5 min at 5000 × g in a 4°C Sorvall 3B. Media was decanted and cells were resuspended in 50 mL of 2XYT containing 100 μg/mL ampicillin. Single-domain antibody expression was induced with 1 mM IPTG and cells grown overnight incubation at 30°C with shaking. Cells were pelleted at 5000 × g for 5 min in a 4°C Sorvall 3B, washed with 30 mL of cold PBS, and collected. Bacterial pellets were then resuspended in 4 mL TBS-T pH 7.5 (0.2% tween) and treated with 60 μL of 500k unit/mL polymyxin B. To facilitate extraction of periplasmic space proteins, cells were incubated at 37°C with shaking for 1 h. Bacterial extracts were clarified by centrifugation at 14,000 × g for 30 min in a 4°C Sorvall 3B. Supernatants were separated from pellets and an additional clarification step was performed in 2 mL Eppendorf tubes at 14,000 × g for 10 min in a 4°C desktop centrifuge. Twice clarified supernatants were added to pre-equilibrated Qiagen Ni-NTA spin columns using a vacuum manifold flow system. Columns were washed once with 600 μL TBS-T containing 10 mM imidazole, and twice with 600 μL TBS-T containing 20 mM imidazole. Spin columns were transferred to collection tubes and protein was eluted with 600 μL TBS-T containing 500 mM imidazole. Eluted protein was dialyzed overnight at 4°C into PBS using 3.5K MWCO snakeskin dialysis tubing (ThermoFisher #88242). Protein concentration was determined using a deNovix DS-11+ spectrophotometer. Purity of recovered J8 was determined by SDS-PAGE analysis using a 16% acrylamide gel. Large scale J8 production was obtained by scaling up to 2-L cultures. Isolated single-domain antibody was loaded on Ni-NTA HiTrap columns using a peristaltic pump. Protein was eluted using an AKTA Pure, then positive fractions were pooled for overnight dialysis. The purified NCATS-J8 (NCATS-BL0252) and NCATS-J8-Avi (NCATS-BL0253) proteins were stored at −20°C until needed.

#### Protein ELISA

Nunc Maxisorp ELISA plates were coated with 50 μL of Aβ40 fibrils or Aβ40 monomer at 5 μg/mL per well in PBS. Plates were incubated overnight at 4°C with rocking. Nanobodies were diluted to 20 μg/mL in PBS, then blocked 1:1 with ELISA phage block (3% milk in Superblock) for 1 h at room temperature. Due to the larger size of donanemab compared to J8 (145.1 kDa vs. 15.4 kDa), a top concentration of 188.4 μg/mL was generated. Plates were blocked in the same fashion after coating solution was removed. Plates had blocking solution removed and subsequently replaced with the 50 μL blocked single-domain antibody or donanemab. Plates were incubated for 1.5 h with gentle rocking at room temperature. Plates were then washed three times with ELISA wash buffer (PBS/Tween (0.05%)). Appropriate wells either had 50 μL secondary 1:1000 diluted anti-HA-HRP or 50 μL secondary 1:1600 diluted anti-Human IgG (H + L)-HRP (#31410), prepared in ELISA phage block, added for 45 min with rocking. Wells were washed 6 times with ELISA wash buffer. Each well had 50 μL of TMB substrate added for 20 min, then reactions were stopped with 50 μL of 0.2 M H_2_SO_4_. The absorbance (450 nm) was determined on a Pherastar plate reader.

#### Biotinylation of the NCATS-J8-Avi single-domain antibody (sdAb)

A biotinylation reaction was prepared by mixing 120 μL of J8-Avi (4.21 mg/mL), 12.6 μL of 200 mM ATP, 2.5 μL of 10 mM Biotin, 1.3 μL of 1M MgSO_4_, 103.6 μL of ultra-pure H_2_O, and 10 μL of BirA ligase supplied in the Enzymatic Protein Biotinylation Kit (Sigma-Aldrich, CS0008). The reaction was incubated on an orbital shaker overnight at 30°C. To maintain protein solubility, 500 μL of pH 7.4 PBS (Gibco #10010-023) was used to dilute protein to approximately 0.667 mg/mL. The reaction mixture was then dialyzed against 2.5 L of pH 7.4 PBS to reduce excess biotin. The post-dialysis concentration of biotinylated J8-Avi of 0.467 mg/mL was determined using λ280 absorbance where 1A = 1 mg/mL on a DeNovix DS-11+ spectrophotometer. Lastly, the biotinylated sdAb was stored at −20°C for downstream applications.

#### Electron microscopy of nanobody- Aβ40 fibril interaction

To prepare labeled nanobody, 500 ng of biotinylated NCATS-J8-Avi sdAb was mixed 1:1 with Alexa FluorTM 488 streptavidin, 10 nm colloidal gold conjugate (Thermo Scientific, A32361) and diluted to a working concentration of 1.2 ng/μL using pH 7.4 PBS. For fibril labeling, 1 μL of 1.2 ng/μL colloidal gold NCATS-J8-AVI complex was mixed with 5 μL of 20 μM freshly prepared Aβ40 fibrils and incubated at room temperature for 15 min. Next, 5 μL of labeled fibrils was adsorbed to glow-discharge carbon-coated grids for 1 min. Grids were blotted dry and subsequently washed for 3 times for 10 s each with ultra-pure water. Lastly, grids were stained with 2% (w/v) uranyl acetate for 30 s and blotted dry. Images were acquired using an Advanced Microscopy Techniques XR-550B camera on an FEI Morgagni microscope, operating at 80 kV.

#### Differentiation of iPSCs into cortical neurons

The procedure was reported previously.^48^ Neuronal differentiation of iPSCs was induced using an induction medium (IM-N2) followed by a neuronal culture medium (CM). IM-N2 contained Knockout DMEM/F12, N2 supplement, NEAA, Gluta-MAX, Chroman I, and doxycycline. On Day 0, iPSCs at appropriate confluency were dissociated with Accutase, centrifuged, resuspended in IM-N2, and seeded onto Matrigel-coated plates. Cells were cultured for four days with daily medium changes containing doxycycline and were monitored for neurite outgrowth. On Day 4, cells with visible neurites were replated onto pre-coated poly-L-ornithine dishes and maintained in CM composed of BrainPhys medium, B27+ supplement, GDNF, BDNF, NT-3, laminin, and doxycycline to support neuronal maturation.

#### Differentiation of iPSCs into forebrain organoids

3D culture and organoid growth were performed as previously described.^49^ iPSCs cultured on Matrigel were dissociated into single cells with Versene and seeded into 12-well AggreWell plates at 4,000 cells per well. On the next day, spheroids were transferred to ultra-low attachment plates and cultured in phase I medium containing DMEM/F12, KnockOut Serum Replacement, GlutaMAX, nonessential amino acids, β-mercaptoethanol, Pen/Strep, dorsomorphine, and A83-01. After five days, the medium was changed to phase II medium composed of DMEM/F12, *N*-2 supplement, GlutaMAX, nonessential amino acids, Pen/Strep, WNT3a, CHIR-99021, and SB-431542. On day 7, spheroids were embedded in Matrigel and cultured for seven additional days. On day 14, spheroids were released from Matrigel and transferred to a SpinΩ bioreactor rotating at 120 rpm in phase III medium containing DMEM/F12, *N*-2 and B-27 supplements, GlutaMAX, nonessential amino acids, β-mercaptoethanol, Pen/Strep, and insulin. After 50 days, cultures were switched to final differentiation medium composed of Neurobasal medium, B-27 supplement, GlutaMAX, β-mercaptoethanol, Pen/Strep, ascorbic acid, cAMP, BDNF, and GDNF. At day 107, organoids were dissociated using a mixture of Accutase and trypsin with DNase I and plated on Matrigel-coated chambered glass slides, or they were fixed and sectioned for immunostaining (Dexrogen Inc). For fibrils and single-domain antibody treatment, day-87 forebrain organoids were cultured on an orbital shaker at 120 rpm and treated with 50 nM fibrils with or without 200 nM J8 co-treatment in final differentiation medium with medium changes every other day. Organoids were collected after 32 days of treatment for sectioning or RNA sequencing.

#### Postmortem AD patient brain sample immunostaining

Brain tissue immunostaining was performed using free-floating sections. All buffers were prepared in advance, including 1× TBS, TBS-A (TBS with 0.1% Triton X-100), TBS-B (TBS-A with 5% BSA), and blocking solution consisting of TBS-B supplemented with 5% normal horse serum. On day 1, sections were washed twice in TBS for 5 min each. Antigen retrieval by citrate buffer incubation at 80°C and formic acid treatment was optional and was omitted in this experiment. Sections were washed twice in TBS, followed by sequential incubations in TBS-A for 15 min and TBS-B for 30 min. Sections were then blocked for 1 h at room temperature in blocking solution. After blocking, sections were transferred carefully into antibody solution (Anti-Beta-Amyloid, 1–16 (6E10) Catalog #803014 [1:2000] and J8 at 200 nM) and incubated overnight at 4°C with gentle agitation in primary antibodies diluted in blocking solution. On day 2, sections were transferred to netwells and washed twice in TBS-A for 5 min, followed by a 15 min wash in TBS-B. Sections were incubated in the dark with Alexa Fluor 555 donkey anti-mouse secondary antibody for 1 h at room temperature, washed three times in TBS, and then incubated with Alexa Fluor 488 mouse anti-HA antibody for 1 h at room temperature. Sections were washed three times in TBS. Autofluorescence quenching was performed by incubating sections for 4 min in freshly prepared 1× TrueBlack solution diluted in 70% ethanol, followed by three washes in TBS. Sections were mounted onto glass slides, coverslipped with DAPI Fluoromount, and allowed to dry overnight in the dark. Coverslips were sealed with nail polish before imaging with Keyence fluorescence microscope at 20 × lense.

#### Image acquisition and processing

Fluorescence confocal images were acquired using a Nikon CSU-W1 SoRa microscope with temperature and CO_2_ control. Image reconstruction, 3D rendering, and time-lapse analysis were performed using Imaris or ImageJ. Fluorescence intensity was quantified with Fiji/ImageJ. Random fields were selected, split into individual channels, and analyzed using a fixed threshold for each channel. Particle analysis was used to identify fluorescent structures and measure size and intensity. Statistical analysis was performed using Excel or GraphPad Prism (versions 9.0). Student’s *t* test or one-way ANOVA was used to calculate *p* values. Curve fitting and IC50 calculations were performed in GraphPad Prism.

#### Single-nucleus RNA-seq data generation and primary processing

Samples from treated brain organoids were pooled, snap-frozen and sent out for sequencing at Novogene Inc. Briefly, Single-nucleus RNA sequencing of human forebrain organoids was performed using the 10× Genomics Chromium platform. 20 brain organoids derived from 3 individual iPSC lines in 2 different batches were pooled per sample condition. Nuclei were isolated from snap-frozen organoid samples and used for generation of 3′ gene expression libraries using the 10× Genomics Single Cell 3′ workflow. Libraries were sequenced on an Illumina NovaSeq X Plus platform (paired-end 150 bp), targeting approximately 75 Gb of raw data per sample and ∼25,000 read pairs per nucleus.

Sequencing data were processed using standard pipelines including demultiplexing, alignment to the human reference genome, cell barcode identification, and UMI counting prior to downstream analysis. Raw sequencing data were processed using the Cell Ranger pipeline (10× Genomics, version 7.0 or later). Base call files were demultiplexed, aligned to the reference genome (e.g., GRCh38/mm10), and gene expression matrices were generated using the cellranger count function. The resulting filtered gene-barcode matrices (filtered_feature_bc_matrix) were used for downstream analyses.

### Quantification and statistical analysis

#### General statistical analysis

Statistical analysis was performed using Excel or GraphPad Prism (version 9.0). Student’s *t* test or one-way ANOVA was used to calculate *p* values, as indicated in the respective figure legends. Curve fitting and IC_50_ calculations were performed in GraphPad Prism. For organoid immunostaining quantification (Figure 4F), five cells were quantified from each of three organoids, cell-level measurements were averaged within each organoid before statistical analysis, and only organoid-level means were treated as independent observations; groups were compared using an ordinary one-way ANOVA followed by Tukey’s multiple-comparisons test. Specific replicate numbers (N) and statistical tests are reported in the corresponding figure legends.

#### Seurat object generation and quality control

Downstream analyses were conducted in R using Seurat v5. Filtered 10× Genomics matrices were imported separately for Control, Fibrils, and FibJ8 using Read10X, and a Seurat object was generated for each library using CreateSeuratObject. Sample, condition, fibril-exposure, and J8-treatment metadata were added, and cell barcodes were prefixed with sample-specific identifiers before the objects were merged.

For each nucleus, the number of detected genes (nFeature_RNA), total UMI count (nCount_RNA), and percentage of mitochondrial transcripts were calculated. Mitochondrial content was computed with PercentageFeatureSet using the human mitochondrial-gene pattern ˆMT-. Pre-filtering violin and scatterplots were examined by sample. Nuclei were retained when they contained more than 200 and fewer than 6,000 detected genes and less than 20% mitochondrial RNA. The upper gene-count threshold provided an initial filter against potential multiplets, whereas the mitochondrial threshold excluded nuclei with evidence of substantial cellular stress or cytoplasmic contamination.

Doublets were identified before integration using scDblFinder. Raw RNA count layers were first joined, converted to a SingleCellExperiment, and analyzed on a per-sample basis using the sample identity supplied to scDblFinder. Doublet classifications and scores were transferred back to the Seurat object by barcode matching, and only nuclei classified as singlets were retained for subsequent analysis.

#### Normalization, cell-cycle scoring, and data integration

Following doublet removal, the data were split by sample. Each sample was log-normalized for cell-cycle scoring, and S-phase and G2/M-phase scores were calculated using the updated Seurat cell-cycle gene sets. Variance stabilization and normalization were then performed independently for each sample using SCTransform, with 3,000 variable features and the “glmGamPoi” method. Mitochondrial transcript percentage, S-phase score, and G2/M-phase score were included as variables to regress during transformation.

The canonical correlation analysis (CCA)-integrated object was carried forward for the reported cluster-marker and annotation analyses. For CCA integration, 3,000 integration features were selected using SelectIntegrationFeatures. The sample-specific objects were prepared using PrepSCTIntegration, integration anchors were identified with FindIntegrationAnchors using SCT normalization, and the data were integrated using IntegrateData. The integrated assay was scaled and subjected to PCA, with 50 principal components calculated. The first 20 principal components were used to construct the nearest-neighbor graph and to perform graph-based clustering at a resolution of 0.5. A two-dimensional t-distributed stochastic neighbor embedding (t-SNE) was computed on the same 20 principal components using Seurat’s RunTSNE function with default perplexity, and nuclei were displayed on the resulting embedding colored by curated cell-class assignment.

The integrated assay was used for dimensionality reduction, clustering, and visualization. Unintegrated RNA counts or log-normalized RNA expression values were used for cluster-marker and condition-associated expression analyses.

#### Cluster-marker identification, cell-type annotation, and curation

Conserved marker genes for each CCA-derived cluster were identified on the joined, log-normalized RNA assay using Seurat’s FindConservedMarkers function. Each cluster was compared with all remaining nuclei using the Wilcoxon rank-sum test, with sample identity (Control, Fibrils, or FibJ8) specified as the grouping variable. Only clusters containing at least 10 nuclei both within and outside the cluster in each sample were tested. Positive conserved markers were retained when they showed an adjusted (p)-value ≤0.05, average log2 fold change >1, and an expression-frequency difference (pct.1 − pct.2) ≥ 0.25 in all three samples, with consistent direction across conditions.

The significant conserved markers were compared with canonical markers reported in the literature, the Human Protein Atlas, and Azimuth reference-mapping predictions. Final identities were assigned based on concordance across these sources and inspection of marker-expression heatmaps, dot plots, and t-SNE distributions. During manual curation, clusters 8, 9, 10, and 12 were excluded, whereas clusters 3 and 5 were merged into a single GABAergic population. The curated clusters were subsequently grouped into the five broad cell classes: excitatory neurons (ExN; *LDB2*, *UNC5C*), inhibitory neurons (InN; *GAD1*, *GAD2*), perivascular cells (*PDGFRB*, *IL33*), oligodendrocyte progenitor cells (OPCs; *OLIG1*, *PDGFRA*), and astrocytes (*GFAP*, *AQP4*).

Cluster-marker analysis was performed using the CCA-derived cluster assignments, whereas gene-expression testing was conducted on the unintegrated RNA assay. For Seurat v5 objects, RNA expression layers were joined using JoinLayers. When a normalized RNA data layer was not already available, expression values were generated using NormalizeData with the LogNormalize method and a scale factor of 10,000.

Conserved marker genes were identified for each CCA-derived cluster using Seurat’s FindConservedMarkers function. For each analysis, nuclei within the selected cluster were compared with all remaining nuclei, and sample identity, Control, Fibrils, or FibJ8, was specified as the grouping variable. Marker testing was performed on the log-normalized RNA assay using the Wilcoxon rank-sum test, and sample-level results were combined using the minimum-(p) method implemented in the metap package. Both positively and negatively enriched conserved markers were evaluated.

Only clusters containing at least 10 nuclei within the cluster and at least 10 nuclei outside the cluster in each of the three samples were included in conserved-marker testing. Candidate markers were required to be detected in at least 10% of nuclei in one of the compared groups. An absolute log2 fold-change threshold of 1 and an absolute difference of 0.25 in the fraction of nuclei expressing the gene within versus outside the cluster were applied during candidate selection. Following marker testing, stringent sample-specific filtering was applied. A positive conserved marker was required to have a Seurat-adjusted (p)-value ≤0.05, an average log2 fold change >1, and a difference in expression frequency of at least 0.25 between the cluster and the remaining nuclei in each of the Control, Fibrils, and FibJ8 samples. Negative conserved markers were retained using the corresponding criteria of an average log2 fold change < −1 and an expression-frequency difference ≤ −0.25 in all three samples. Marker direction was therefore required to be consistent across all experimental conditions.

Initial positive markers for the CCA-derived clusters were identified using FindAllMarkers. Marker testing was performed on the joined, log-normalized RNA assay using the Wilcoxon rank-sum test, with each cluster compared against all remaining nuclei. Candidate positive markers were required to be detected in at least 25% of nuclei in one of the compared groups and to have a minimum average log2 fold change of 0.25. Seurat-adjusted *p*-values below 0.05 were used to prioritize marker genes. These tests were used for cluster characterization and annotation rather than for inferential comparisons between experimental treatments.

Conserved markers were ranked using the minimum absolute log2 fold change and minimum absolute expression-frequency difference observed across the three samples, followed by the maximum sample-specific adjusted (p)-value. The highest-ranking positive conserved markers from each cluster were carried forward for cell-type annotation and for visualization using marker-expression heatmaps and dot plots. This marker analysis was used for cluster characterization and annotation and was not interpreted as treatment-associated differential-expression testing.

The top-ranked positive conserved markers for each curated cluster were visualized as an expression heatmap. For plotting, the joined RNA assay was log-normalized (NormalizeData, LogNormalize method, scale factor 10,000), and the selected marker genes were scaled and centered across nuclei (z-scored per gene). Scaled expression values were displayed using Seurat’s DoHeatmap, with nuclei grouped by curated cluster identity and marker genes ordered by their associated cluster. Color intensity represents normalized (z-scored) expression, clipped to the range shown in the color scale (approximately −2 to +2). The heatmap was generated from the unintegrated, log-normalized RNA assay.

#### Excitatory-neuron directional-reversal and pathway enrichment analysis

Nuclei annotated as excitatory neurons were selected from the final Seurat object. Because each condition was represented by one pooled 10× library, individual nuclei were not treated as independent biological replicates, and cell-level FindMarkers/Wilcoxon *p*-values were not used for condition-associated inference. Raw RNA counts were aggregated within each condition-level library to form pseudobulk profiles and normalized to log2 counts per million (log2CPM). Genes were retained if they were protein-coding (excluding unannotated ENSG-only identifiers, lincRNAs, pseudogenes, and chromosome-Y genes) and met an expression floor of mean CPM ≥5 across the Control, Fibrils, and FibJ8 libraries, applied to limit unstable log-ratios from low-count genes.

For each retained gene, descriptive effects were calculated as ΔF = log2CPM(Fibrils) − log2CPM(Control) and ΔJ = log2CPM(FibJ8) − log2CPM(Fibrils). Genes were assigned to quadrants by the signs of these effects: Q1 (+,+), Q2 (−,+), Q3 (−,−), and Q4 (+,−). Effect-qualified genes were defined by |ΔF| ≥ 0.10 and |ΔJ| ≥ 0.10, yielding 2,319 genes. Q2 genes (suppressed with fibrils, elevated with J8) were designated candidate directional-reversal genes and ranked by reversal strength, defined as |ΔJ|. The J8 response was also visualized after stratifying genes by the direction of the fibril-associated effect; no inferential test was applied to this gene-level distribution.

Candidate directional-reversal genes (pooled Q2 and Q4) were carried forward for KEGG pathway and Gene Ontology Biological Process enrichment using ShinyGO v0.85 (FDR ≤0.05). All effects are descriptive summaries from single pooled libraries per condition and do not establish biological-replicate-level treatment effects or transcriptomic rescue.

#### Independent cross-study comparison

Published excitatory-neuron differential-expression summaries were obtained from Leng et al.^32^ for entorhinal-cortex Braak stage 2 versus 0 and Braak stage 6 versus 0 comparisons and from Morabito et al.^33^ for Alzheimer’s disease versus control. For the Leng dataset, gene-level log-fold changes were summarized as the median across reported excitatory-neuron subpopulations, with the minimum reported FDR and global FDR retained. For the Morabito dataset, excitatory-neuron rows were selected and duplicate gene entries were summarized using the median average log-fold change and minimum adjusted *p*-value. Gene symbols were standardized to uppercase, and genes absent from an external table were treated as missing rather than as having zero effect.

Gene-level concordance was evaluated among genes present in both the organoid and external datasets. Each shared gene was classified as directionally concordant when the non-zero organoid Fibrils-versus-Control effect and the external disease effect had the same sign. For each comparison, we report the number of shared genes and the percentage of directionally concordant genes, and tested this percentage against the 50% value expected by chance using a two-sided exact binomial test. Directional concordance was evaluated independently of statistical-significance thresholds; for prioritization of externally supported genes, a global FDR ≤0.10 was applied.

For the cross-study heatmap, candidate FibJ8 directional-reversal genes identified in the organoid-only analysis were required to show opposite Fibrils and FibJ8 effects (|log2FC| ≥ 0.2) and were ranked by the number of external datasets supporting the Fibrils-associated disease direction, followed by directional-reversal strength and the absolute organoid Fibrils-versus-Control effect. Common sex-associated genes were omitted from the default ranking. Because effect-size scales differed across studies, values within each comparison were converted to signed ranks ranging from −1 to 1, preserving both effect direction and relative magnitude. The external datasets supported only the Fibrils-associated disease direction and did not independently validate the FibJ8 response.

For pathway-level comparisons, Reactome and Gene Ontology Biological Process gene sets were obtained from MSigDB using msigdbr. Preranked gene set enrichment analysis was performed separately for the organoid Fibrils-versus-Control effect vector and each external disease-effect vector using fgseaMultilevel. Gene sets containing 10–500 genes were tested, with eps = 1 × 10^−10^; GSEA was performed when at least 500 ranked genes were available. Pathway concordance was evaluated by comparing normalized enrichment scores (NES) between the organoid and each external dataset, with pathways classified as concordant when the organoid and external NES had the same sign, and the percentage of concordant pathways reported for each comparison. The configured pathway FDR threshold was 0.10, although directional concordance was evaluated independently of statistical significance.

All cross-study analyses were performed after the organoid-only Fibrils and FibJ8 analyses. They were interpreted as descriptive assessments of disease-direction consistency and do not replace independent biological replication or establish treatment efficacy.

#### Technical characterization and reproducibility considerations

Organoids were generated from three independent iPSC lines across two differentiation batches.

## Supplemental information

Document S1. Figures S1–S4

Table S1. Cluster marker genes identified in single-nucleus RNA sequencing of human forebrain organoids, related to Figure 4

Table S2. Excitatory-neuron directional-reversal genes, pathway enrichment, and cross-study concordance with human Alzheimer disease datasets, related to Figures 5–7 (A) Directional-reversal genes across Control, Fibrils, and FibJ8 conditions, including expression, annotation, filtering, and external-dataset support metrics. (B) GO biological process and KEGG enrichment of directional-reversal genes. (C) Gene-level concordance between Fibrils versus Control and the Leng Braak 2, Leng Braak 6, and Morabito AD datasets. (D) GO biological process pathway concordance with Leng Braak 2 versus 0. (E) Reactome pathway concordance with Leng Braak 2 versus 0. (F) GO biological process pathway concordance with Leng Braak 6 versus 0. (G) Reactome pathway concordance with Leng Braak 6 versus 0. (H) GO biological process pathway concordance with Morabito AD versus Control. (I) Reactome pathway concordance with Morabito AD versus Control.
