# Supplementary material for: A fibril-selective single-domain antibody targets pathogenic amyloid-β assemblies and protects human neurons
Source: iScience. 2026 Oct 21;29(11):117946. doi: 10.1016/j.isci.2026.117946 (PMC13718768; doi:10.1016/j.isci.2026.117946)
Supplement: Supplementary file 1 [file mmc1.pdf]

iScience, Volume *29*

Supplemental information

A fibril-selective single-domain antibody targets pathogenic amyloid-β assemblies and protects human neurons

Matt A. Greenlee, Soukaina Amniouel, Isabel Rivera, Jessica Suh, Natalia J. Martinez, Anton Simeonov, Elizabeth Head, Robert Tycko, Wei Zheng, Bryan D. Fleming, and Qi Zhang

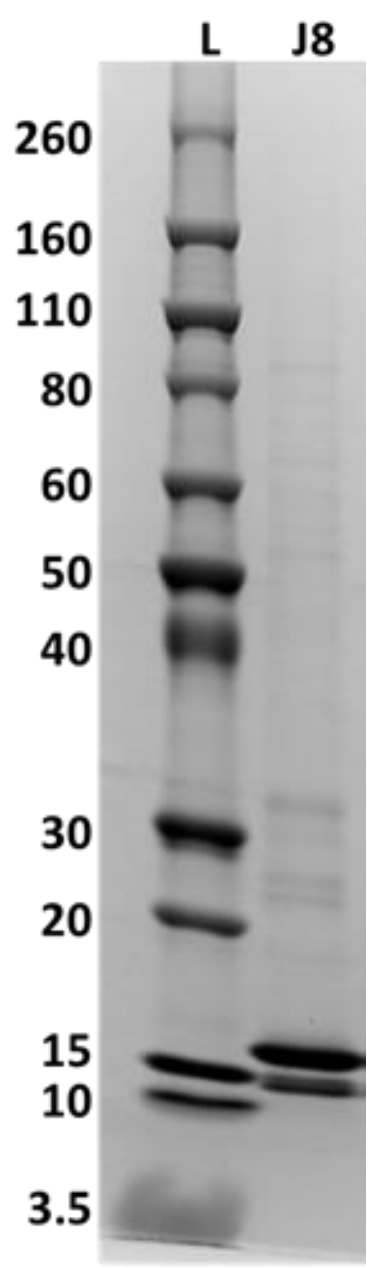

Figure S1. Purity of NCATS-J8 assessed by SDS-PAGE, related to Figure 1. Lanes were loaded with 10 µl of Novex Sharp pre-stained protein ladder or 1 µg of single-domain antibody under non-reducing conditions. Protein was visualized with Thermo Fisher Imperial protein stain.

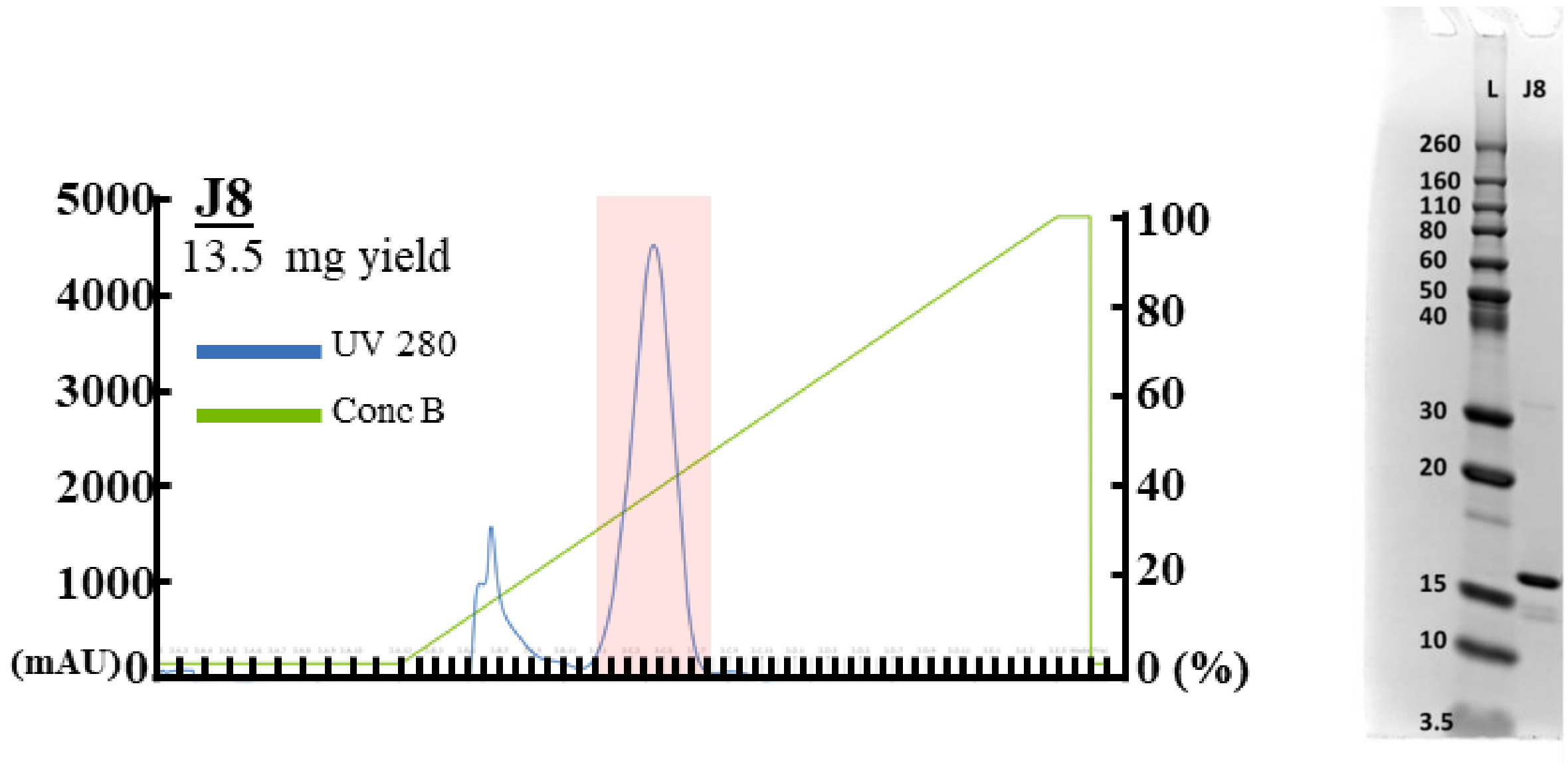

Figure S2. Large-scale purification of NCATS-J8, related to Figure 1. ÄKTA chromatogram showing the elution profile of J8 purification, with purity of the recovered single-domain antibody assessed by SDS-PAGE. Lanes were loaded with 10 µl of Novex Sharp pre-stained protein ladder or 1 µg of single-domain antibody under non-reducing conditions. Protein was visualized with Thermo Fisher Imperial protein stain.

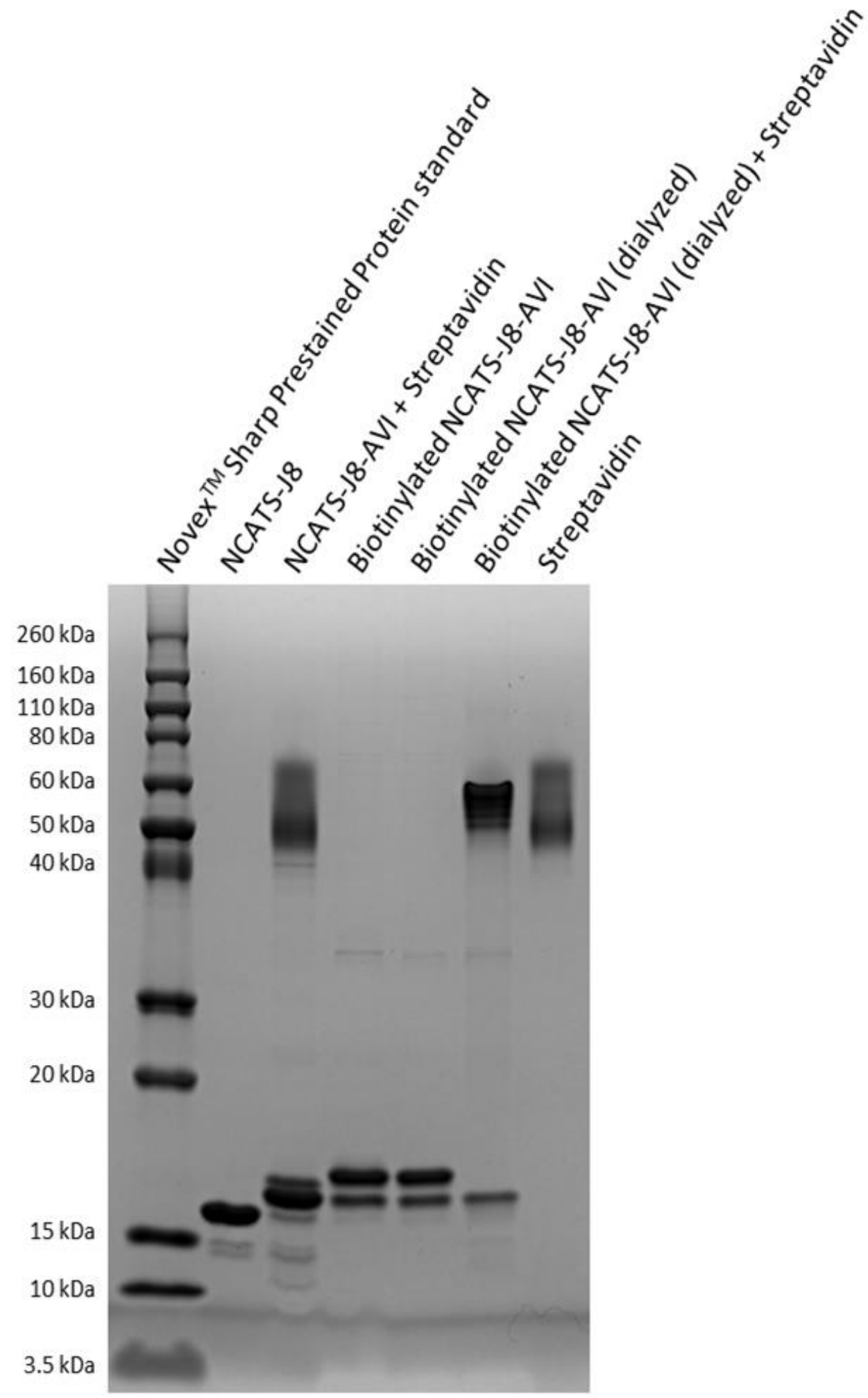

Figure S3. Validation of NCATS-J8-Avi biotinylation by SDS-PAGE mobility shift, related to Figure 1. To assess biotinylation efficiency, 2 µg each of NCATS-J8, NCATS-J8-Avi, biotinylated NCATS-J8-Avi, dialyzed biotinylated NCATS-J8-Avi, dialyzed biotinylated NCATS-J8-Avi combined with 2 µg streptavidin, and 2 µg streptavidin alone were resolved on a 16% polyacrylamide gel.

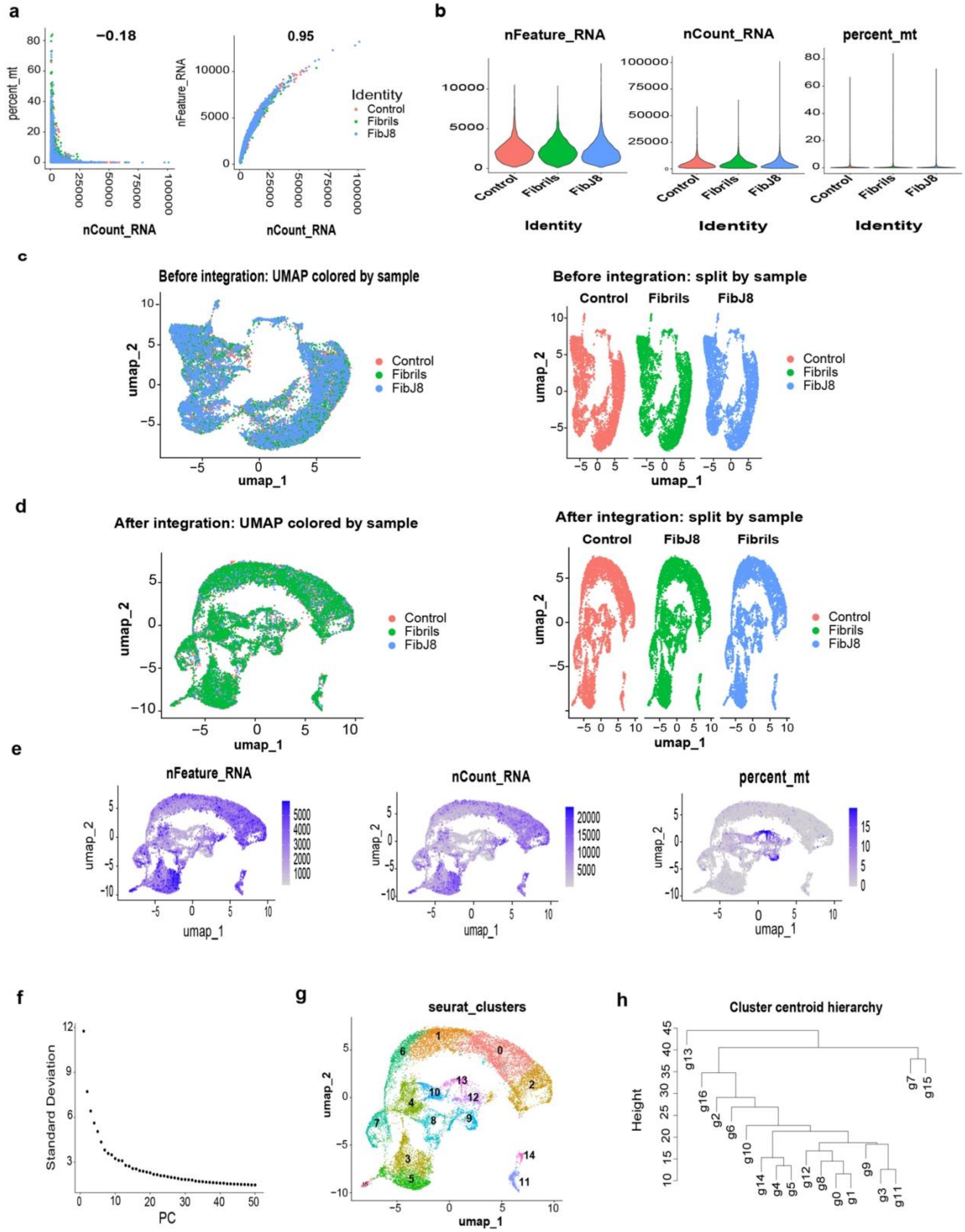

Figure S4. Quality control, integration, and clustering of single-nucleus RNA-sequencing data, related to Figure 4. (A) Scatter plots showing relationships between quality-control metrics,

including mitochondrial gene percentage (percent.mt) versus total RNA counts (nCount_RNA) and detected genes (nFeature_RNA), used to assess cell quality. (B) Violin plots showing the distribution of nFeature_RNA, nCount_RNA, and percent.mt across samples (Control, Fibrils, and FibJ8) prior to filtering. (C) UMAP visualization of cells before dataset integration, colored by sample (left) and split by sample (right), highlighting batch effects between datasets. (D) UMAP visualization after integration, colored by sample (left) and split by sample (right), showing improved alignment of cells across samples. (E) Feature plots showing the distribution of quality metrics (nFeature_RNA, nCount_RNA, and percent.mt) across the integrated UMAP space. (F) Elbow plot used to determine the number of principal components selected for downstream analysis. (G) UMAP of the integrated dataset showing transcriptionally defined clusters. (H) Hierarchical clustering dendrogram illustrating relationships among identified cell clusters based on transcriptomic similarity.
